# Maternal antibodies induced by a live attenuated vaccine protect neonatal mice from cytomegalovirus

**DOI:** 10.1038/s41541-023-00602-4

**Published:** 2023-02-03

**Authors:** Vu Thuy Khanh Le-Trilling, Andreja Jagnjić, Ilija Brizić, Mareike Eilbrecht, Kerstin Wohlgemuth, Carmen Rožmanić, Alan Herdman, Katja Hoffmann, Astrid M. Westendorf, Hartmut Hengel, Stipan Jonjić, Mirko Trilling

**Affiliations:** 1 Institute for Virology, University Hospital Essen, University of Duisburg-Essen, Essen, Germany; 2 Center for Proteomics, Faculty of Medicine, University of Rijeka, Rijeka, Croatia; 3 Institute of Virology, Medical Center, Faculty of Medicine, University of Freiburg, Freiburg, Germany; 4 Institute for Medical Microbiology, University Hospital Essen, University of Duisburg-Essen, Essen, Germany

**Keywords:** Viral infection, Experimental models of disease

## Abstract

Human cytomegalovirus (HCMV) frequently causes congenital infections, resulting in birth defects and developmental disorders. A vaccine is needed, but unavailable. We analyzed the potential of CMV mutants, lacking their STAT2 antagonists to serve as live attenuated vaccine viruses in mice. Infections with attenuated viruses elicited strong ELISA-reactive binding IgG responses and induced neutralizing antibodies as well as antibodies stimulating cellular Fcγ receptors, including the antibody-dependent cellular cytotoxicity (ADCC)-eliciting receptors FcγRIII/CD16 and FcγRIV. Accordingly, vaccinated mice were fully protected against challenge infections. Female mice vaccinated prior to gestation transmitted CMV-specific IgG to their offspring, which protected the progeny from perinatal infections in a mouse model for congenital CMV disease. To define the role of maternal antibodies, female mice either capable or incapable of producing antibodies were vaccinated and subsequently bred to males of the opposite genotype. Challenge infections of the genotypically identical F1 generation revealed the indispensability of maternal antibodies for vaccine-induced protection against cytomegaloviruses.

## Introduction

Human cytomegalovirus (HCMV) is the prototypical member of the β-*herpesvirinae*. The majority of the adult global population is latently infected with HCMV. Worldwide, the seroprevalence of HCMV is 30–100%, depending on factors such as age, gender, and socioeconomic conditions (see e.g., refs. ^1,2^). In the majority of cases, the healthy adult immune system efficiently keeps HCMV in check. However, if the immune system is impaired, senescent, or not yet sufficiently developed, HCMV infections are not properly controlled and can cause life-threatening diseases. The latter case is particularly relevant since HCMV is frequently transmitted from the pregnant mother through the placenta to the developing fetus^3^. In terms of the number of children with long-term sequelae and childhood fatalities, congenital CMV (cCMV) outnumbers several other well-known childhood disorders such as Down syndrome, Spina Bifida, and fetal alcohol syndrome^4^. Thus, women of childbearing age would particularly benefit from a vaccine against HCMV. However, despite relevant progress, an approved HCMV vaccine is neither available nor in sight (comprehensively reviewed in ref. ^5^). The genetic heterogeneity of clinical HCMV isolates suggests that HCMV super-infections can occur^6–9^. Therefore, it is a matter of debate whether sterilizing immunity to CMV infection represents an achievable goal for a vaccine. However, it is evident that even vaccines with low efficacy can have substantial epidemiological and economic benefits^10^. Additionally, concepts such as vaccination against disease and population-based vaccinations may render imperfect vaccines clinically relevant—either by alleviating the incidence and/or severity of disease^11^ or by slowing virus dissemination^12^.

One immediate immune response crucial to survive viral infections is the production of interferons (IFNs)^13^. IFNs are cytokines that induce a very potent direct antiviral state and orchestrate adaptive immunity, including antibody responses^14^. Upon binding of IFNs to the cognate receptors, *signal transducer and activator of transcription* (STAT) proteins are activated. These transcription factors induce the expression of adjacent IFN-stimulated genes (ISGs). Besides IFN-dependent gene induction, our recent data revealed the existence of a class of IFN-repressed genes (IRepGs)^15,16^. ISGs and IRepGs together establish a potent antiviral state. However, during the long co-evolution with their hosts, CMVs developed potent antagonists of the IFN system^17–22^. HCMV and mouse cytomegalovirus (MCMV) encode the analogous interferon antagonists pUL145 and pM27, respectively, which mark STAT2 for proteasomal degradation by misappropriation of cellular Cullin RING ubiquitin ligases^23–28^.

MCMV mutants lacking *M27* coding capacity (ΔM27-MCMV) are highly IFN susceptible in vitro^25,27,28^ and highly attenuated in vivo^27–29^. Despite the pronounced attenuation of ΔM27-MCMV, we observed low but consistent virus replication at 3 days post-infection in the spleen and the liver of infected C57BL/6, BALB/c, and 129 mice^27^. Since it was shown that viral replication of a live attenuated MCMV vaccine is a prerequisite for the induction of protective immunity^30,31^, we hypothesized that ΔM27-MCMV, despite its pronounced attenuation, may produce sufficient antigens and immune stimulation to mount protective immune responses (e.g., neutralizing and Fcγ receptor-activating antibodies). As most viruses code for antagonists of the IFN system, the analysis of ΔM27-MCMV, an attenuated virus mutant lacking an IFN antagonist, might serve as a model system for the attenuation of other viruses. Due to the fact that cCMV is one of the most relevant HCMV-associated diseases and that maternal immunoglobulins are transmitted from mother-to-child, we focused our attention here on humoral immune responses.

## Results

### An attenuated MCMV mutant lacking the STAT2 antagonist expresses viral antigens and stimulates cytokine responses

**Fig. 1:**
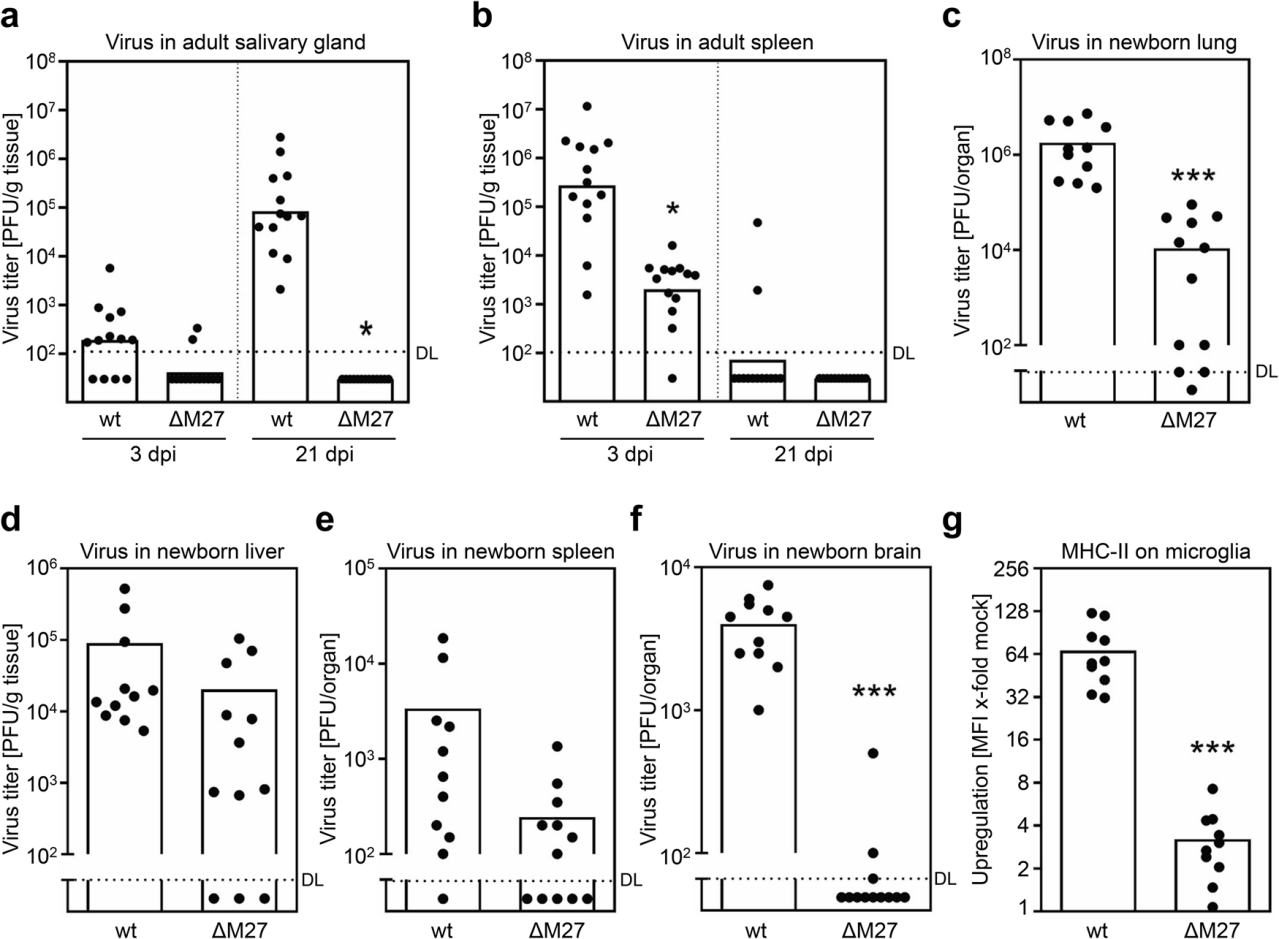
ΔM27-MCMV replicates to low levels at early times post-infection. BALB/c mice were infected i.p. with wt-MCMV or ΔM27-MCMV. At 3 and 21 days post-infection (dpi), the indicated organs were collected and frozen. The virus titers were determined from organ homogenates by plaque titration. Titrations were performed in quadruplicates. Pooled data of three independent experiments are shown. Bars depict the geometric mean, dots show the titers of individual mice (*n* = 13). DL detection limit. **a** Virus titer in the salivary gland. **b** Virus titer in spleen. **c**–**f** Newborn C57BL/6 mice were i.p. injected with 500 PFU of WT- or ΔM27-MCMV at ~24 h postpartum, and viral titers were determined in indicated organs at 9 dpi by plaque assay. Titers of individual mice (dots) and median values (bars) are shown (*n* = 11–12). **g** As in C, but surface expression of MHC-II by live CD45^int^ CD11b^+^ microglia cells was determined by cytometry. The gating strategy is shown in Supplementary Fig. 1. Median fluorescence intensity (MFI) values of MHC-II expression were normalized to mock control samples (*n* = 10). Significance was calculated by heteroscedastic unpaired one-tailed *t*-test. *p < 0.05. ***p < 0.001.

MCMV mutants lacking the IFN inhibitor-encoding gene *M27* (ΔM27-MCMV) replicate largely normal in fibroblast cell cultures, but are severely attenuated in vivo^28,29^ as a result of the inability to antagonize STAT2-dependent IFN signaling^27^. Accordingly, no replication of ΔM27-MCMV was detectable in the salivary glands at 21 days post-infection (dpi), while all wt-MCMV-infected mice exhibited high MCMV titers (Fig. 1a). Nevertheless, low-level and transient ΔM27-MCMV replication occurred in the spleen early after infection (Fig. 1b). A very stringent test for the attenuation of MCMV mutants is the assessment of virus replication in newborn mice, and in particular the dissemination to the brain in these mice. Therefore, newborn C57BL/6 mice were infected 24 h postpartum with MCMV and viral titers were determined at 9 dpi. ΔM27-MCMV replication was significantly reduced (*t*-test *p* < 0.001) in the lungs of newborn mice (Fig. 1c). In livers and spleens, we also observed lower titers of ΔM27-MCMV as compared to wt-MCMV (Fig. 1d, e). Most importantly, the replication in the brain was significantly reduced (*t*-test *p* < 0.001), and only detectable at all in 3 out of 12 mice (Fig. 1f). One hallmark of the MCMV infection of the brain and the corresponding inflammation is the pronounced upregulation of major histocompatibility complex class II molecules (MHC-II) on the surface of microglia cells^32^. To address this parameter, we isolated leukocytes from the brain and stained MHC-II on CD45^int^ CD11b^+^ microglia cells. As expected, wt-MCMV caused a very prominent upregulation of MHC-II on microglia cells, which was significantly reduced (*t*-test *p* < 0.001) in the case of ΔM27-MCMV infection (Fig. 1g and Supplementary Fig. 1). We concluded that the deletion of the STAT2 antagonist *M27* leads to a pronounced attenuation of MCMV in vivo in adult as well as in the more vulnerable newborn mice.

To investigate if the low-level replication of ΔM27-MCMV is sufficient to stimulate immunity, we first analyzed the early innate immune responses raised by ΔM27-MCMV in adult mice. We collected serum samples at 3 and 21 dpi and determined the concentration of the cytokines TNFα, IL-6, CXCL10/IP-10, CCL5/RANTES, CCL4/MIP-1β, CXCL1, IL-12p70, IL-1β, and IL-33 by Luminex assay. TNFα, IL-6, CXCL10/IP-10, CCL5/RANTES, and CCL4/MIP-1β serum concentrations were significantly increased as compared to naïve control animals (see Table 1, left panel). No significant differences concerning cytokine induction were observed when wt-MCMV and ΔM27-MCMV-infected mice were compared at 3 dpi. At 21 dpi, all analyzed cytokine levels returned back to baseline levels comparable to naïve animals (Table 1, right panel). TNFα levels were slightly elevated at 21 days post ΔM27-MCMV infection compared to wt-MCMV, but did not differ from naïve animals. Cytokines such as CCL5/RANTES, CXCL10/IP-10, and CCL4/MIP-1-β are well known for their immuno-stimulatory and immune cell-attractive capacities, suggesting that ΔM27-MCMV not only expresses antigens, as indicated by the detection of replicating virus, but also induces a cytokine milieu that may recruit and activate immune cells.

**Table 1:** ΔM27-MCMV stimulates cytokine responses.

Cytokine	naive mean ± SD	wt 3 dpi mean ± SD	ΔM27 3 dpi mean ± SD	ΔM27 vs Mock 3 dpi *p* value	ΔM27 vs wt 3 dpi *p* value	wt 21 dpi mean ± SD	ΔM27 21 dpi mean ± SD	ΔM27 vs Mock 21 dpi *p* value	ΔM27 vs wt 21 dpi *p* value
TNFα	0.59 ± 0.44	4.29 ± 3.96	2.85 ± 0.47	0.0004	NS	0.54 ± 0.11	0.81 ± 0.13	NS	0.02
IL-6	2.89 ± 1.39	47.64 ± 55.89	14.11 ± 2.01	0.0002	NS	2.19 ± 0	1.82 ± 0.9	NS	NS
CXCL10/IP-10	19.39 ± 8.51	778.54 ± 743.95	384.84 ± 43.22	0.0003	NS	18.72 ± 3.82	20.24 ± 6.11	NS	NS
CCL5/RANTES	137.95 ± 102.59	414.73 ± 432.59	380.19 ± 65.45	0.0101	NS	64.26 ± 15.78	96.53 ± 32.93	NS	NS
CCL4/MIP-1β	6.20 ± 0	334.18 ± 267.75	217.63 ± 36.61	0.0014	NS	6.20 ± 0	6.20 ± 0	ND	ND
CXCL1	116.60 ± 158.67	224.56 ± 218.38	171.86 ± 78.98	NS	NS	26.84 ± 6.26	42.61 ± 17.59	NS	NS
IL-12p70	5.81 ± 6.36	20.17 ± 18.34	10.22 ± 0	NS	NS	2.07 ± 0.65	2.96 ± 0.66	NS	NS
IL-1β	11.07 ± 4.14	10.26 ± 1.45	9.63 ± 1.26	NS	NS	9.63 ± 1.26	11.70 ± 3.91	NS	NS
IL-33	44.09 ± 30.45	26.15 ± 29.44	23.81 ± 14.80	NS	NS	34.42 ± 23.29	54.59 ± 19.99	NS	NS

Concentration [pg/ml] Mean ± standard deviation (SD); NS not significant (*p* > 0.05); ND not determined.

Serum samples of naïve, wt-MCMV-, and ΔM27-MCMV-infected BALB/c mice (*n* = 4 per group) were collected at 3 and 21 dpi. The levels of indicated cytokines from serum samples were quantified using Luminex technology. The table shows mean and standard deviation. Significance was calculated by heteroscedastic unpaired two-tailed *t*-test.

### An MCMV mutant lacking the IFN antagonist pM27 induces MCMV-specific and neutralizing antibodies

**Fig. 2:**
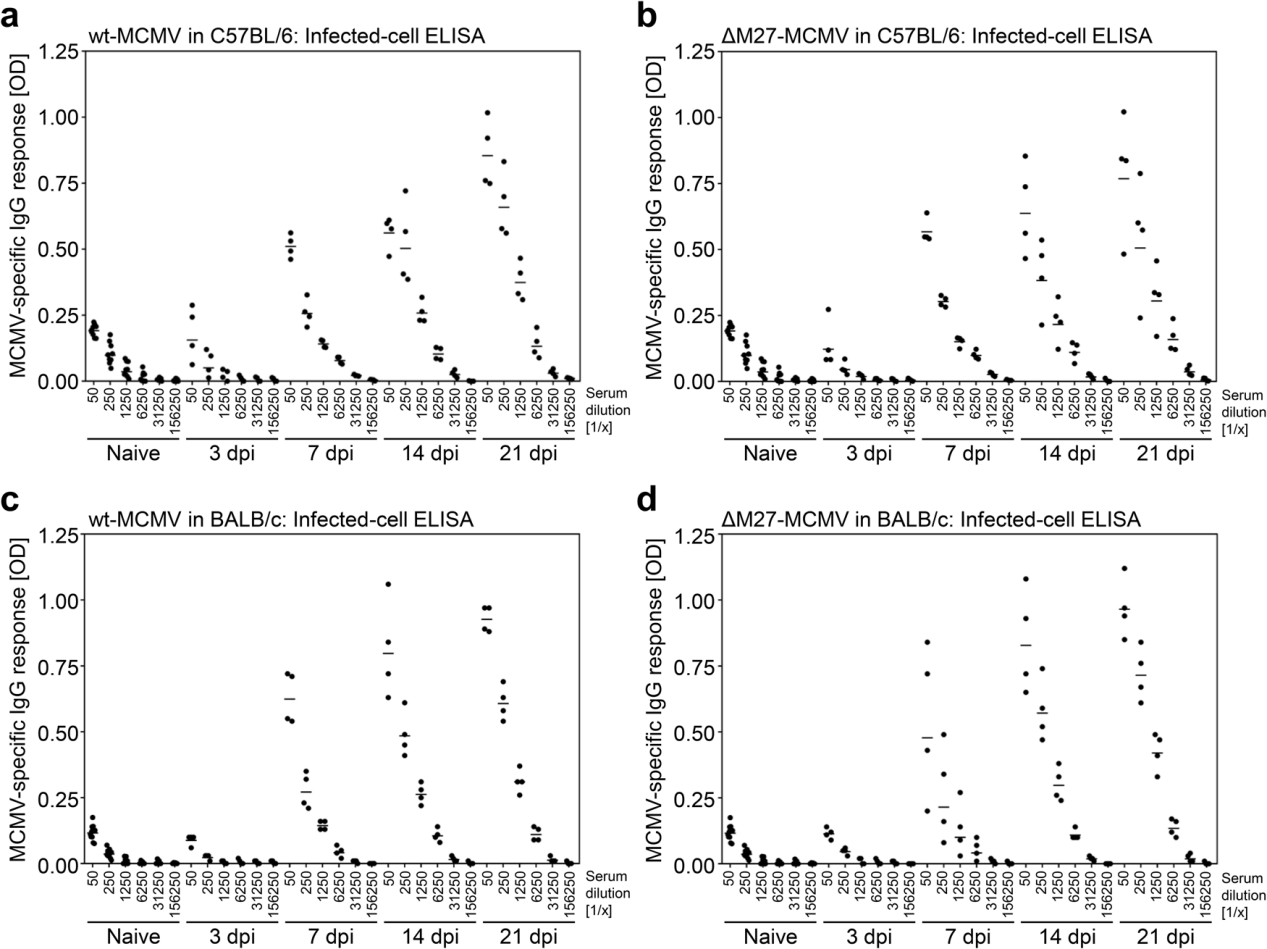
Immunization with ΔM27-MCMV induces MCMV-specific antibodies recognizing infected cell proteins. C57BL/6 (**a**, **b**) and BALB/c (**c**, **d**) mice were infected i.p. with wt-MCMV or ΔM27-MCMV. At 3, 7, 14, and 21 dpi, serum samples were collected. MCMV-specific IgG antibodies recognizing MCMV-infected cell proteins were quantified by ELISA using indicated serum dilutions. All ELISA measurements were performed in triplicates. Shown is the mean for each mouse. Sera of naïve animals served as control. The geometric mean values (horizontal bars) as well as the values of individual mice (*n* = 4 per group) are shown.

**Fig. 3:**
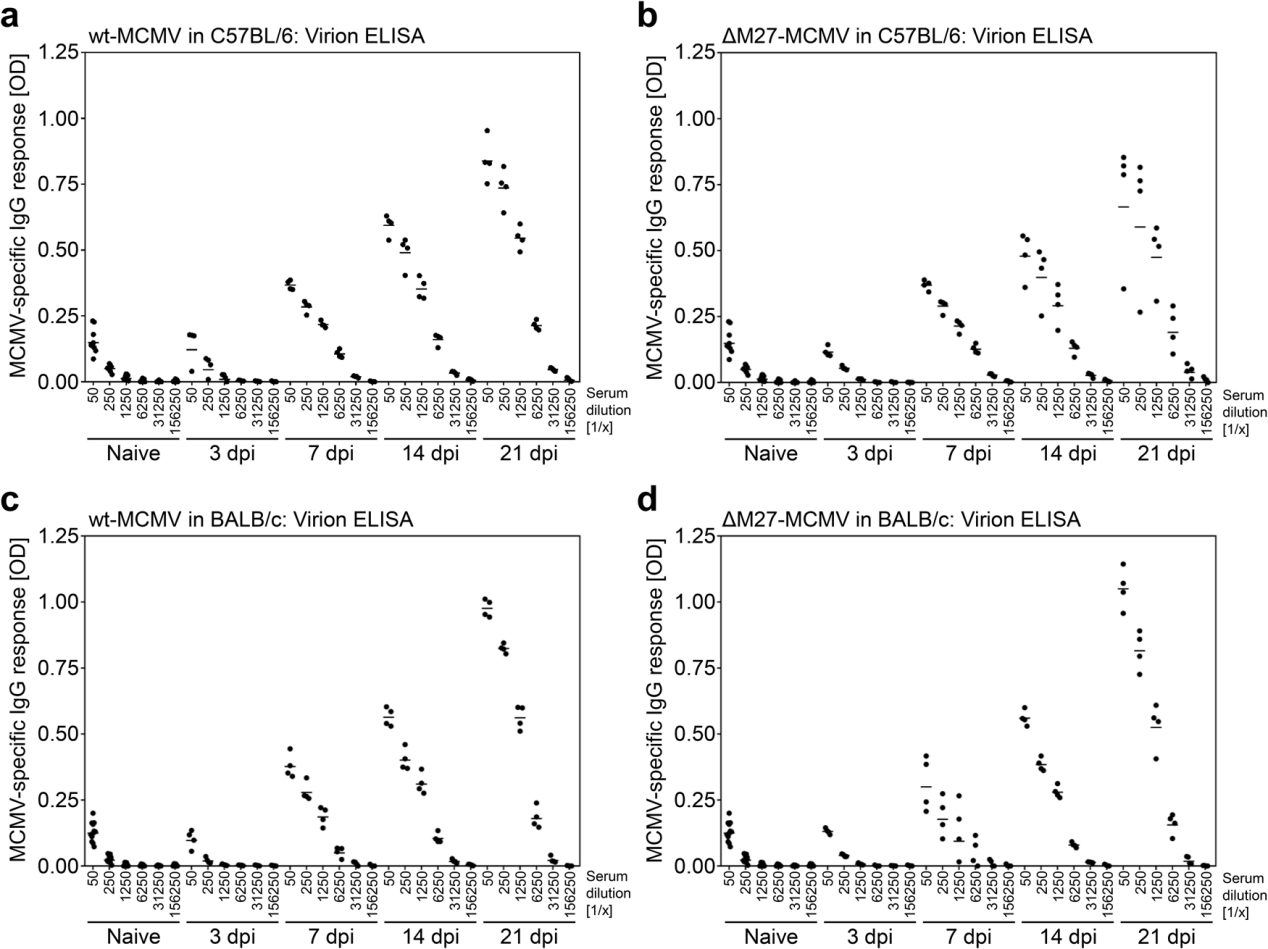
Immunization with ΔM27-MCMV induces MCMV-specific antibodies recognizing MCMV particles. C57BL/6 (**a**, **b**) and BALB/c (**c**, **d**) mice were infected i.p. with wt-MCMV or ΔM27-MCMV. At 3, 7, 14, and 21 dpi, serum samples were collected. MCMV-specific IgG antibodies recognizing purified MCMV particles were quantified by ELISA using indicated serum dilutions. ELISA measurements were performed in triplicates. Shown is the mean of each mouse. Sera of naïve animals served as control. The geometric mean values (horizontal bars) as well as the values of individual mice (*n* = 4 per group) are shown.

**Fig. 4:**
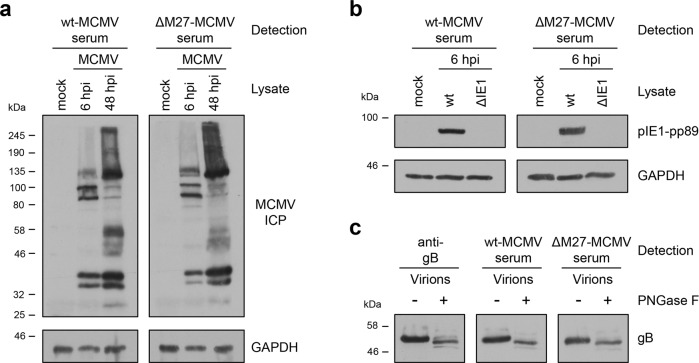
Immunization with ΔM27-MCMV elicits antibody responses similar to wt-MCMV infection in terms of quantity and antigens recognized. BALB/c mice were infected i.p. with wt-MCMV or ΔM27-MCMV. At 21 dpi, serum samples were collected. Serum samples of 10 wt-MCMV and 10 ΔM27-MCMV-infected mice were pooled and used for the analysis of recognized MCMV antigens. **a** Mouse fibroblasts were infected with wt-MCMV. At 6 and 48 hpi, cells were lysed. **b** Mouse fibroblasts were infected with ΔIE1-MCMV^88^ and the corresponding wt-MCMV control virus. At 6 hpi, cells were lysed. **c** Virus particles from the supernatant of wt-MCMV-infected fibroblasts were concentrated by ultracentrifugation through a 15% sucrose cushion and lysed. Lysates of concentrated virus particles were mock-treated or digested with PNGase F. **a**–**c** Immunoblot analyses were performed using serum samples of wt-MCMV or ΔM27-MCMV-infected mice for detection of MCMV proteins. Uncropped scans are provided in Supplementary Fig. 2.

**Fig. 5:**
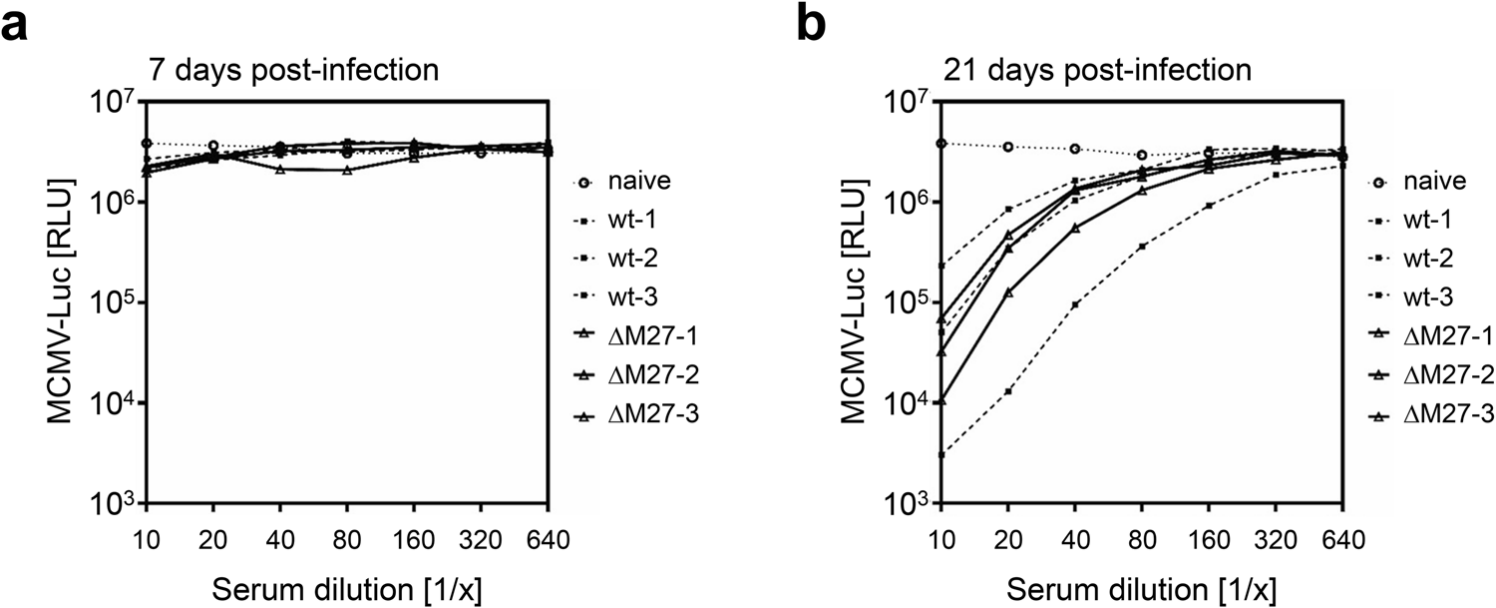
Immunization with ΔM27-MCMV elicits neutralizing antibody responses. BALB/c mice were infected i.p. with wt-MCMV or ΔM27-MCMV. At 7 and 21 dpi, serum samples were collected. Different dilutions of serum samples were incubated with MCMV:Luc for 90 min prior to infection of fibroblasts. At 24 h post-infection, cells were lysed and luciferase activity was measured. **a** Neutralizing capacity of serum samples collected at 7 dpi. **b** Neutralizing capacity of serum samples collected at 21 dpi.

Based on the antigen expression and cytokine induction, we tested if ΔM27-MCMV may be able to mount an adaptive humoral immune response. We infected C57BL/6 mice with wt-MCMV or ΔM27-MCMV and collected serum samples at 3, 7, 14, and 21 dpi to quantify MCMV-specific immunoglobulin G (IgG) by ELISA. Both viruses induced very strong MCMV-specific ELISA-reactive IgG responses (Fig. 2a, b). This humoral immune response was also evident in BALB/c mice (Fig. 2c, d) that exhibit an increased MCMV susceptibility due to lacking expression of the activating natural killer (NK) cell receptor Ly49H. Additionally, ΔM27-MCMV—like wt-MCMV—induced MCMV-specific IgG recognizing purified MCMV particles in C57BL/6 and BALB/c mice (Fig. 3a–d). To determine whether sera collected from wt-MCMV-infected and ΔM27-MCMV-vaccinated animals recognize similar antigens, immune sera of mice infected with wt-MCMV or ΔM27-MCMV were applied to comparatively probe membranes containing MCMV antigens derived from MCMV-infected fibroblasts. Very similar patterns were observed when comparing wt-MCMV and ΔM27-MCMV immune serum-reactive immunoblot signals (Fig. 4a). Using ΔIE1-MCMV and concentrated virus particle preparations, we identified pIE1-pp89 and gB as MCMV proteins recognized by immune sera originating from wt-MCMV infection and ΔM27-MCMV vaccination (Fig. 4b, c and Supplementary Fig. 2). Together, these data indicate that ΔM27-MCMV infection elicits antibody responses similar to wt-MCMV infection in terms of quantity and antigens recognized. Further analysis revealed that the antibodies present after 21 days of ΔM27-MCMV and wt-MCMV infection also exhibit neutralizing capacities (Fig. 5). Thus, the low level and temporary replication of ΔM27-MCMV is sufficient to induce humoral immune responses in terms of IgG antibodies recognizing viral antigens present in MCMV-infected cells and purified MCMV particles (such as pIE1-pp89 and gB) as well as neutralizing antibodies.

### Immunization with ΔM27-MCMV raises strong MCMV-specific FcγR-activating IgG responses

**Fig. 6:**
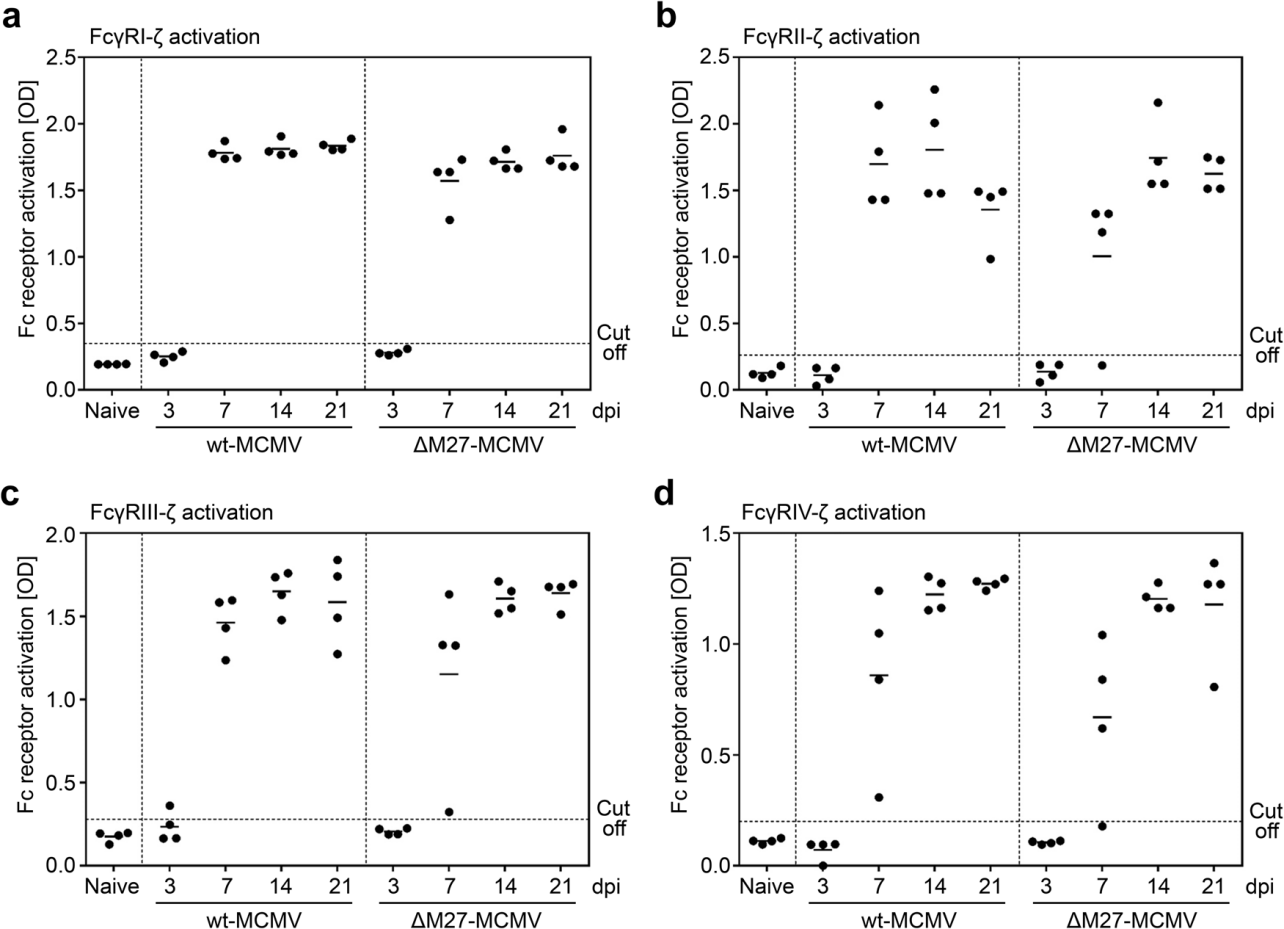
Immunization of BALB/c mice with ΔM27-MCMV raises strong MCMV-specific FcγR-activating IgG responses. BALB/c mice were infected i.p. with wt-MCMV or ΔM27-MCMV. At 3, 7, 14, and 21 dpi, serum samples were collected. Fcγ receptor-activating capacity was quantified from sera by mIL-2 ELISA using a surrogate assay as described in the M&M section and in reference ^37^. The mean values as well as the values of individual mice (*n* = 4 per group) are shown. **a** Activation assay for mFcγRI. **b** Activation assay for mFcγRII. **c** Activation assay for mFcγRIII. **d** Activation assay for mFcγRIV.

The IgG molecules fulfill various biological functions. Traditionally, neutralizing antibodies (nAbs) are considered to be very relevant in terms of virus control. However, recent studies document that other IgG effector functions mediated by cellular Fcγ receptors (FcγRs) also significantly contribute to the protective capacities of IgG^33–35^. Therefore, we applied a previously described test principle^36,37^ to determine the FcγR-activating abilities of the MCMV-specific IgG raised upon immunization with ΔM27-MCMV. Within this assay, IL-2 secretion is a convenient surrogate for FcγR activation by immune complexes composed of IgG and the cognate antigen (see Supplementary Fig. 3 for a schema). Starting at 7 days after immunization of BALB/c mice, ΔM27-MCMV induced strong in vivo IgG responses that activate FcγRI/CD64 (Fig. 6a), FcγRII/CD32 (Fig. 6b), FcγRIII/CD16 (Fig. 6c), and FcγRIV (Fig. 6d). These responses were comparable to the ones induced by wt-MCMV (Fig. 6a–d). The analysis of more resistant C57BL/6 mice showed comparable results (Supplementary Fig. 4). Taken together, these data indicate that immunization with a live attenuated MCMV mutant lacking a STAT2 antagonist elicits IgG responses that activate immune cells through Fcγ receptors.

### Vaccination with ΔM27-MCMV elicits immune responses that protect adult mice from a subsequent challenge infection

**Fig. 7:**
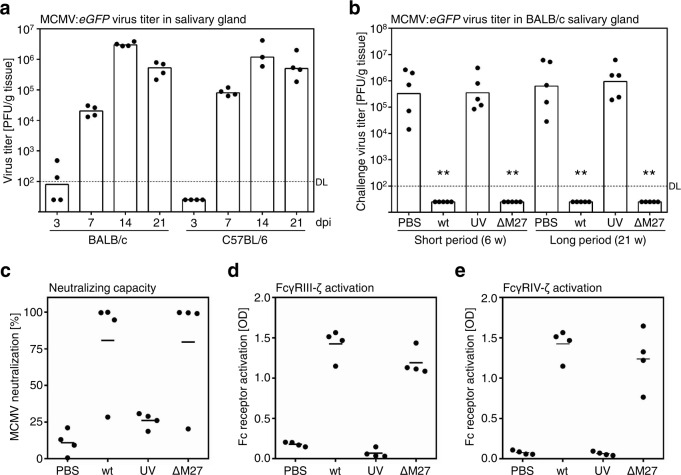
Vaccination with ΔM27-MCMV protects adult mice from challenge infections. **a** C57BL/6 and BALB/c mice were infected i.p. with MCMV:eGFP. At 3, 7, 14, and 21 dpi, salivary glands were harvested and frozen (*n* = 3-4 per group). The virus titers were determined from organ homogenates by plaque titration. **b** BALB/c mice were vaccinated with 2 × 10^5^ PFU of wt-MCMV, ΔM27-MCMV, or UV-inactivated wt-MCMV. At 6 and 21 weeks post vaccination, mice were challenged with 2 × 10^5^ PFU of MCMV:eGFP. At 21 days post-challenge infection, salivary glands were harvested and frozen. The MCMV:eGFP titers were determined from organ homogenates by plaque titration. All titrations were done in quadruplicate. Bars depict the geometric mean, dots show titers of individual mice (*n* = 4-5 per group). DL detection limit. All vaccinated groups were compared to the corresponding control group by Kruskal–Wallis test corrected for multiple comparisons by controlling the false discovery rate. **p value <0.01. ***p value <0.001. **c**–**e** Neutralizing antibody titers and Fcγ receptor-activating capacity were quantified from serum samples collected one week prior to the challenge infection (5 weeks after vaccination). The mean values as well as the values of individual mice are shown. Protection of vaccinated animals are shown in Fig. 7b. **c** In vitro neutralization assay. **d** Activation assay for mFcγRIII. **e** Activation assay for mFcγRIV.

Given the induction of humoral immune responses by immunization with ΔM27-MCMV, we tested its vaccine potential in adult mice. To differentiate the immunization and challenge virus inoculum, we used the eGFP-expressing MCMV strain RVG102^38^ (“MCMV:eGFP”) as challenge virus. The most consistent and reliable replication of MCMV in BALB/c and C57BL/6 mice is observed in the salivary gland (SG) (see e.g., ref. ^27^). Persistent and recurrent shedding from the SG is believed to be the principal means by which MCMV spreads in the population^39^. Since we aimed to challenge the vaccinated animals with MCMV:eGFP, we determined its in vivo replication in the SG. Peak SG titers were observed for both BALB/c and C57BL/6 strains at 14 dpi and remained high at 21 dpi (Fig. 7a). We decided to assess the MCMV titers at 21 dpi in the following vaccination experiments to exclude misinterpretations in terms of vaccine efficacy based on delayed replication. Since it is well known that preexisting immunity strongly reduces the peak titers of subsequent MCMV infections^30,40,41^, we used the wt-MCMV-induced protection as a positive control for our experiments. We started the vaccination experiments in C57BL/6 mice due to their intrinsic resistance against MCMV, which is based on the recognition of the viral protein m157 by Ly49H. When these mice were challenged at 6 weeks after vaccination, we observed full protection of mice infected with wt-MCMV or vaccinated with ΔM27-MCMV (Supplementary Fig. 5, left panel). In clear contrast, vaccination attempts with UV-irradiated MCMV particles failed to restrict replication (Supplementary Fig. 5), indicating that a non-replicating virus is not sufficient to raise protective immune responses. To analyze if the protection is long-lasting, a second group of mice was challenged at 21 weeks after vaccination. Again, vaccination with ΔM27-MCMV, but not with a UV-inactivated MCMV, mediated full protection (Supplementary Fig. 5, right panel). In order to address the question if susceptible mice can also be protected by vaccination with a live attenuated MCMV lacking a STAT2 antagonist, we recapitulated the experiments in BALB/c mice. All mice that received wt- or ΔM27-MCMV efficiently controlled the challenge MCMV infection (Fig. 7b). This protection correlated with the presence of humoral immune responses which was analyzed one week before the challenge (5 weeks after vaccination) in the very same animals (Fig. 7c–e). As already observed for C57BL/6 mice, none of the vaccinated BALB/c mice exhibited detectable SG MCMV replication irrespective of whether the animals were vaccinated for 6 or 21 weeks before the challenge (Fig. 7b). As described above, we applied the eGFP-expressing MCMV strain RVG102 for the challenge. To exclude that this leads to exaggerated protection, e.g., due to an attenuation caused by the introduction of the *eGFP* gene, we repeated the ΔM27-MCMV vaccination and challenged with wt-MCMV. We observed full protection of ΔM27-MCMV-immunized mice against this wt-MCMV challenge (Supplementary Fig. 6). Interestingly, dose deescalating studies indicated that vaccination doses below 10^4^ PFU of ΔM27-MCMV were fully protective (Supplementary Fig. 7A). When we analyzed the antibodies induced by immunization with low doses of ΔM27-MCMV, we detected MCMV-specific IgG responses similar to the vaccination with higher doses (Supplementary Fig. 7B). Taken together, we concluded that low doses of an attenuated MCMV vaccine lacking an IFN antagonist elicit immune responses, which provide long-term protection against challenge infections in resistant and susceptible mice.

### Vaccinated female mice transmit MCMV-specific IgG and protection to their offspring

**Fig. 8:**
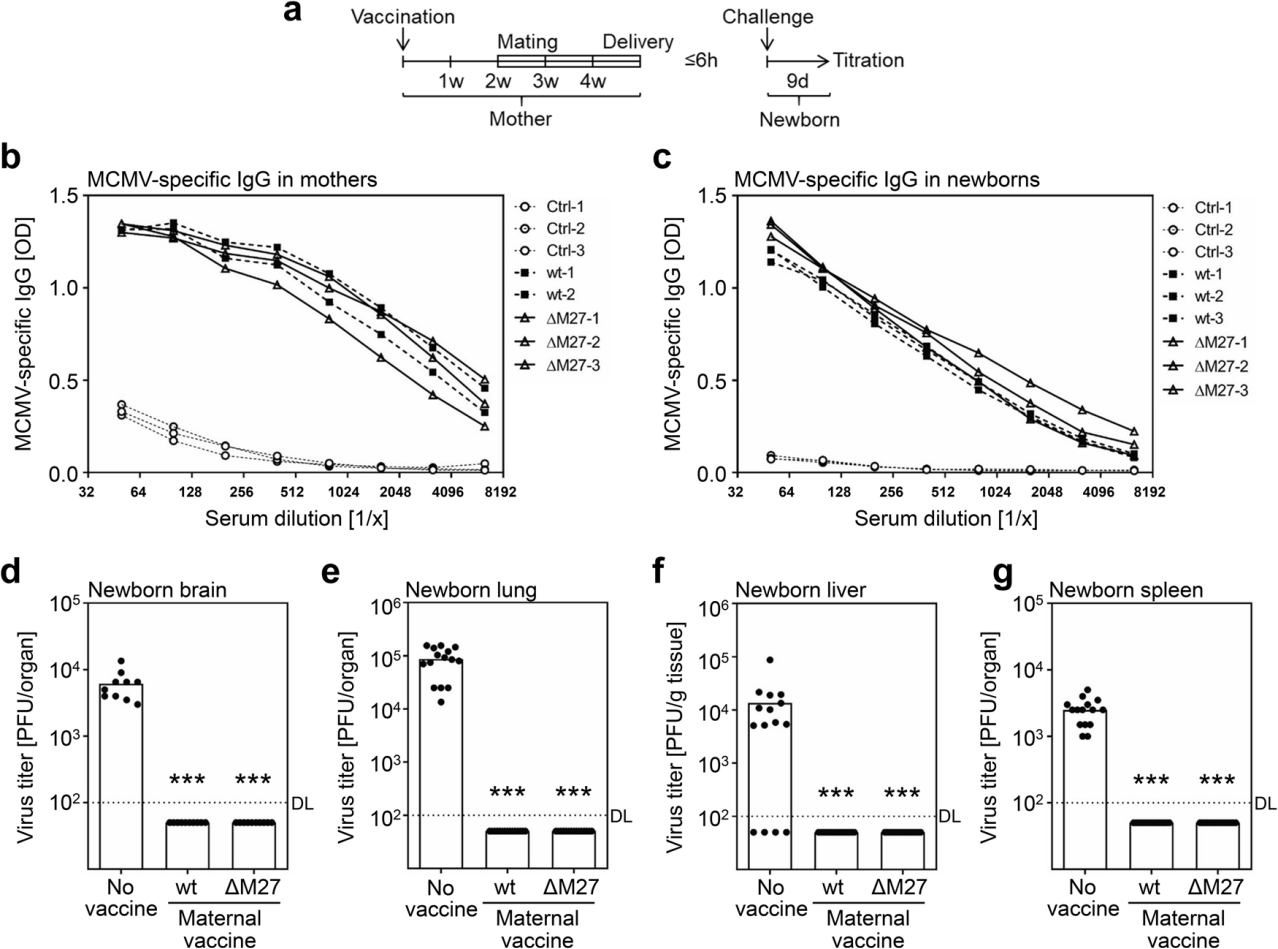
Vaccinated female mice transmit MCMV-specific IgG and protection to their offspring. Female BALB/c mice were vaccinated with wt-MCMV or ΔM27-MCMV. At 5 weeks post vaccination, mice were mated to analyze the protection of the offspring. At ≤6 h postpartum, groups of five newborns derived from three dams (*n* = 5 × 3) were challenged i. p. with 500 PFU of MCMV:eGFP per mouse. At 9 d post challenge, the indicated organs were harvested and virus titers were determined by plaque titration. **a** Schema of the experimental design. The presence of MCMV-specific IgG was tested by ELISA for dams (**b**) and their offspring (**c**). ELISA reactivity of different dilutions of serum samples was analyzed. Values of individual serum dilutions and individual mice are shown. **d** Virus titers of brains of pups. **e** Virus titers of lungs of pups. **f** Virus titers of livers of pups. **g** Virus titers of spleens of pups. Bars depict the geometric mean, dots show the titers of individual mice. DL detection limit. All vaccinated groups were compared to the corresponding control group by Kruskal–Wallis test corrected for multiple comparisons by controlling the false discovery rate. ***p value <0.001.

Congenital HCMV infections are responsible for a high burden of disease to the population. Although cCMV also occurs in women with preexisting immunity^6,42^, there are various indications that maternal IgG responses reduce the likelihood of cCMV or at least alleviate its consequences^43–49^. Based on the strong humoral immune responses raised by vaccination with ΔM27-MCMV, we assessed whether MCMV-specific IgG is transmitted from the vaccinated dam to the offspring. For this purpose, female mice were vaccinated and mated 2 weeks later (Fig. 8a). The presence of MCMV-specific IgG was determined after gestation for both dams and offspring. Strong MCMV-specific ELISA-reactive IgG responses were evident for the mothers (Fig. 8b) as well as the newborn mice (Fig. 8c), indicating efficient transmission of maternal IgG. This raised the question, if vaccinated mothers could transmit sufficient humoral immunity to influence the outcome of the congenital infection. Unfortunately, one limitation of rodent CMV models (such as MCMV and RCMV) is that transplacental infections do not occur. By intraperitoneal MCMV infection of newborn mice during the first hours (<6 h) of life, the groups of Bill Britt and Stipan Jonjić established a model system, which recapitulates central aspects of congenital HCMV infections^50^ and allows the assessment of vaccine efficacies^51^. Applying this model, the protective capacity of ΔM27-MCMV vaccination in terms of transmitted maternal immunity was tested. Three groups of five newborns derived from three different dams (*n* = 5 × 3) were challenged intraperitoneally with 500 PFU of MCMV:eGFP per mouse. At 9 dpi, viral replication was determined in the brain, lung, liver, and spleen of the pups (Fig. 8d–g). Intriguingly, vaccination of the mothers with ΔM27-MCMV prior to gestation conferred complete protection to the newborn mice against the challenge infection.

### Maternal antibodies are indispensable for the mother-to-child transmitted protection

**Fig. 9:**
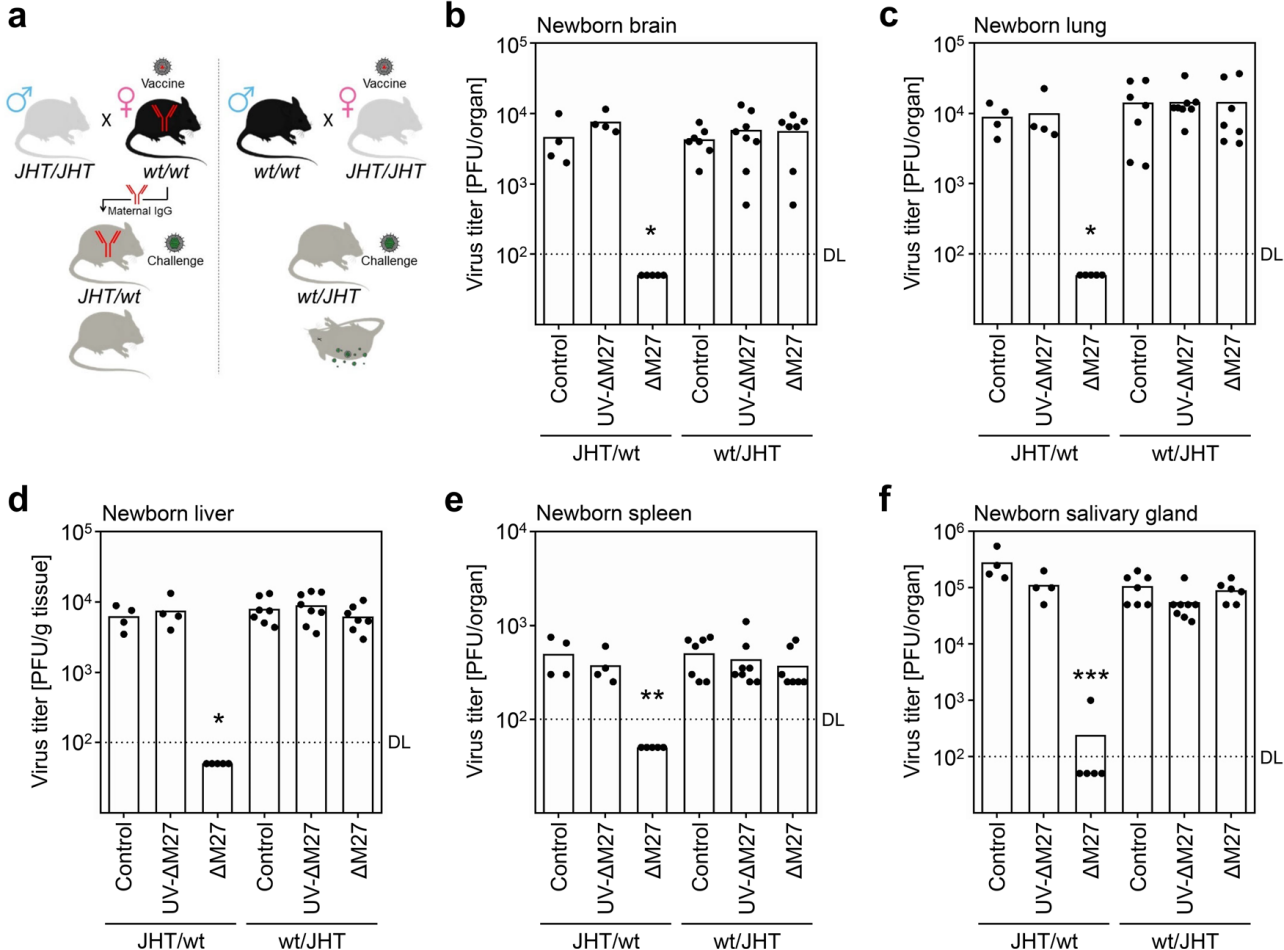
Maternal antibodies are indispensable for mother-to-child transmitted protection. Female C57BL/6 and C57BL/6^JHT/JHT^ (JHT) mice were vaccinated with UV-irradiated ΔM27-MCMV (ΔM27-UV), ΔM27-MCMV (ΔM27) or mock vaccinated (control). At 2 weeks post vaccination, C57BL/6 female mice were mated with C57BL/6^JHT/JHT^ males and C57BL/6^JHT/JHT^ female mice with C57BL/6 males. To analyze the protection of the offspring, at ~24 h postpartum, newborns were challenged i. p. with 500 PFU of MCMV:eGFP per mouse. At 9 d post challenge, the indicated organs were harvested and virus titers were determined by plaque titration. wt and JHT indicate the genotype of dams. **a** Schema of the experimental design. **b** Virus titers of brains of pups. **c** Virus titers of lungs of pups. **d** Virus titers of livers of pups. **e** Virus titers of spleens of pups. **f** Virus titers of the salivary gland of pups. Bars depict the geometric mean, dots show titers of individual mice (*n* = 4–8 per group). DL detection limit. All vaccinated groups were compared to the corresponding control group by Kruskal–Wallis test corrected for multiple comparisons by controlling the false discovery rate. *p value <0.05. **p value <0.01. ***p value <0.001.

The transmission of MCMV-specific IgG from the vaccinated dams to their offspring together with the transfer of protection implied involvement of maternal IgG. But other aspects of the maternal adaptive immune system may also contribute to the observed protection of the neonates. In mammals with a hemochorial placenta (e.g., primates and rodents), the blood-placenta barrier separates the maternal from the fetal bloodstream, limiting the exchange of cells. However, mother-to-child transmission of cells including lymphocytes has been documented^52^. To define the role of maternal antibodies in protecting the offspring of vaccinated mothers, JHT mice were analyzed, in which the deletion of the J elements of the immunoglobulin heavy chain locus (JHT) leads to B cell deficiency. Female mice either capable (Fig. 9a, wt/wt) or incapable (Fig. 9a, JHT/JHT) of producing antibodies were vaccinated with ΔM27-MCMV and subsequently bred to males of the opposite genotype. The genotypically identical F1 generation (JHT/wt or wt/JHT) was challenged with MCMV:eGFP after birth. The virus titers of the brain, lung, liver, spleen, and salivary gland of the newborns demonstrated that only the offspring of vaccinated mothers capable of producing antibodies was protected (Fig. 9b–f). This result provides evidence for the indispensability of maternal antibodies for vaccine-induced mother-to-child transmitted protection.

Altogether, our data indicate that attenuated CMV mutants lacking a STAT2 antagonist raise protective humoral immune responses, which can be transmitted from mother-to-child, and have the potential to serve as live attenuated vaccines.

## Discussion

We show here that a highly attenuated CMV mutant lacking a specific inhibitor of IFN-induced STAT2 signaling elicits strong humoral immune responses and confers full protection against subsequent challenge infections in adult mice. Further, it induces transferable immunity mediated by maternal IgG passed from female mice that had been vaccinated prior to gestation to their unvaccinated offspring. Protective IgG immune responses were raised despite a more than 100-fold reduced virus replication that even prevents the mutant from reaching the salivary gland—the organ which is thought to be indispensable for MCMV dissemination in natura.

Like MCMV, HCMV expresses a protein that induces STAT2 degradation, pUL145^23,25,26^. Both, pM27 and pUL145 interact with STAT2 and exploit DDB1-containing Cullin RING ubiquitin ligases (CRL) to stimulate proteasomal degradation of STAT2^25,26,53^. Our MCMV data presented here suggest that an HCMV mutant lacking its STAT2 antagonist pUL145 may constitute a very interesting candidate for the development of a live attenuated HCMV vaccine. Based on the intriguing findings concerning the potential of cytomegaloviral vectors for vaccination against other infectious agents such as retroviruses (see e.g., refs. ^54,55^), and our work in the mouse^56^, the herein described attenuation principle might also be applicable for the design of CMV-based vaccine vectors.

Depending on the organ, ΔM27-MCMV replicates 10- to 1000-fold less efficient compared to wt-MCMV (Fig. 1 and refs. ^27,28^). Nevertheless, humoral immune responses in terms of binding, neutralizing, and ADCC-eliciting antibodies were almost identical to wt-MCMV-induced IgG responses (Figs. 2–6, 8). Even very low infectious doses of ΔM27-MCMV raised protective immunity (Supplementary Fig. 7), and ΔM27-MCMV vectors expressing hepatitis B virus (HBV) antigen sHBsAg induce superior heterologous protection in the hydrodynamic injection mouse model compared to pM27-expressing parental viruses^56^. Another intriguing side finding in this regard was the apparent discrepancy in vaccine efficacy between UV-inactivated virus and ΔM27-MCMV. None of the mice exhibited measurable protection after receiving UV-irradiated MCMV particles corresponding to 2 × 10^5^ PFU before UV-irradiation. Thus, it seems that the immune system responds more vigorously to replicating MCMV compared to inactivated MCMV virions. The concept of “vita-PAMPs” has been proposed^57^, which suggests that the innate immune system senses certain PAMPs that arise only during the active life cycle of the pathogen. Other explanations for the difference between low levels of replicating virus and vaccination with inactivated virions may be caused by different kinetics of antigen exposure^58^, the involvement of immuno-stimulatory danger-associated molecular patterns (DAMPs) released by infected or dying cells, and distinct antigen compositions of virus particles and infected cells since MCMV virions lack several non-structural proteins^59^.

In the past, several experimental vaccinations with live attenuated herpesviruses including cytomegaloviruses conferred protection in their corresponding rodent models^60–62^. Conversely, even the best clinical vaccine studies in humans remained only partially or transiently successful^63–67^. Why do we anticipate that our current strategy is more promising than previous attempts? We are convinced that the type and degree of attenuation is a key determinants. Virus mutants lacking multiple virulence factors may have an increased safety profile, but may be “over-attenuated” and too weak to sufficiently challenge the immune system. In this regard, it should be emphasized that HCMV vaccine trials using the HCMV strains AD169 or Towne employed HCMVs that had lost the pentameric entry complex^68^, most of the UL*b’* region, which comprises various regulators of immune responses^69–71^, and that had acquired relevant loss-of-function mutations (e.g., affecting RL5A, RL13, UL36, and UL131A in AD169, and RL13 and UL130 in Towne^72^). Additionally, only low virus doses (e.g., 5000 PFU per individual) were applied as vaccine^65^. Opposite to such attempts, we propose to apply a reasonably higher dose of a replication-competent CMV that is attenuated through the lack of a single IFN antagonist.

Recently, a conditionally replication-defective HCMV vaccine named V160 has been evaluated in phase I (NCT01986010^73^ and NCT03840174) and phase 2 (NCT03486834) clinical trials. V160 is a genetically engineered HCMV-AD169 strain expressing the pentameric entry complex, and harboring two chemically controlled protein stabilization switches that render two essential viral proteins prone to rapid degradation. Based on these changes, V160 only replicates in the presence of the synthetic compound Shield-1, and is replication defective in vivo^74^. Due to its RL duplication, V160 lacks the UL*b’* region comprising *UL145*. Thus, even replicating V160 should be unable to degrade STAT2—like our ΔM27-MCMV vaccine. However, in clear contrast to our vaccine, V160 is unable to replicate in vivo. It will be interesting to see if such an inactive HCMV vaccine elicits sufficient immuno-stimulatory capacity to raise protective immunity in women and their babies.

A vaccine that prevents the burden of fetal and newborn disease is highly desirable. Although concerns regarding the feasibility of a CMV vaccine are raised due to observations showing that maternal CMV sero-positivity does not fully protect against cCMV, increasing evidence indicates that maternal immunity does confer a considerable measure of protection against both CMV transmission and CMV disease in newborns (reviewed in ref. ^75^). Our results showed that the transmission of protective humoral immune responses from dams, which were vaccinated prior to pregnancy, to their offspring is achievable, and that maternal IgG is a correlate of protection. Consistent with this interpretation, it was recently shown that maternal antibodies recognizing HSV-1 are transmitted to the offspring, and protect neonatal mice against HSV-1 neurological infection and death^76–78^. Accordingly, live attenuated guinea pig CMV (GPCMV) vaccines improve the pregnancy outcome in the congenital GPCMV infection model^79–82^, and passive immunization with GPCMV-specific antibodies decreased fetal infection, intrauterine growth retardation, and reduced pregnancy losses^83^. Together, these findings suggest that IgG may be the primary protective arm of the immune system in the control of congenital herpesvirus infections. Despite the fact that ΔM27-MCMV is clearly attenuated in adult and neonatal mice (Fig. 1), we would not suggest giving such live attenuated vaccines during pregnancy or to newborns, rather offer them prior to pregnancy to women who intend to have children.

Taken together, we showed that the low-level replication of an attenuated MCMV mutant lacking the ability to antagonize STAT2-dependent IFN signaling is efficient in raising substantial IgG responses, comprising neutralizing and Fcγ receptor-activating antibodies. By using Fcγ receptor-deficient mice, further analyses may dissect whether the protection is mediated by the neutralizing capacity, Fcγ receptor-activating antibodies, or a combination of both. Prophylactic immunization with ΔM27-MCMV acted as an effective vaccine for adult mice and led to the passive transfer of protective immunity mediated by maternal IgG to the offspring. These findings suggest that HCMV mutants lacking the pM27-analogous STAT2 antagonist pUL145 deserve to be further investigated concerning their potential to serve as live attenuated vaccines.

## Methods

### Cells, viruses, and virus titration

Primary mouse fibroblasts (mouse embryonic fibroblasts [MEF] and mouse newborn cells [MNC]) were isolated from mouse embryos and newborns, respectively, according to described protocols^84^. Briefly, day 16–17 post-coitum embryos or 1–2 days old newborn mice were dissected and minced, washed with PBS, and treated with trypsin and DNase I. FCS was added to stop the trypsin reaction before cells were washed and seeded into cell culture flasks. Reporter cells for Fcγ receptor activation were generated by stable transfection of the mouse BW5147 thymoma cell line (ATCC TIB-4)^36,37^. Fibroblasts were grown in DMEM supplemented with 10% (v/v) FCS, 100 µg/ml streptomycin, 100 U/ml penicillin, and 2 mM glutamine. The mouse BW5147 thymoma cell line (ATCC TIB-4) and transfectants thereof were maintained in RPMI 1640 medium containing 10% (v/v) FCS, 100 U/ml penicillin, 100 µg/ml streptomycin, 2 mM glutamine, and 1 mM sodium pyruvate. BW5147:FcγR-ζ transfectants were selected in RPMI medium containing 3 mg/ml G418 (Sigma-Aldrich) or 50 μg/ml Zeocin (Invitrogen). All cell culture media and supplements were obtained from Gibco/Life technologies. The wt-MCMV was reconstituted by transfection of BAC-DNA (MCMV-BAC described in ref. ^85^) into permissive cells. ΔM27-MCMV, MCMV:eGFP, and Δm157-MCMV:Luc are established mutants^25,27,38^. Viral titers were determined by standard plaque titration^86^ on primary MEF or MNC^84^. All in vitro infections and titrations were conducted with centrifugal enhancement (900 g for 30 min). For in vivo infections, mice were infected intraperitoneally with 2 × 10^5^ PFU MCMV per mouse, if not indicated differently. Organs of infected mice were harvested, snap-frozen in liquid nitrogen, and stored at −80 °C until titrations were performed.

### Animal care

C57BL/6 and BALB/c mice were obtained from Charles River or Harlan and either housed in the animal facility of the Institute for Virology of the University Hospital Essen or housed and bred under specific pathogen-free conditions at the Central Animal Facility, Faculty of Medicine, University of Rijeka. Mice were maintained under selective pathogen-free conditions.

### Ethics statement

All procedures were performed in accordance with the guidelines of the University Hospital Essen, Germany, the national animal protection law (Tierschutzgesetz [TierSchG]), and the recommendations of the Federation of European Laboratory Animal Science Association (FELASA). The study was approved by the Northrhine-Westphalia State Office for Nature, Environment, and Consumer Protection (LANUV NRW, Düsseldorf, Northrhine-Westphalia, Germany; permit #84-02.04.2014.A390 for MEF preparation and #84-02.04.2013.A414 for MCMV in vivo infections of adult mice) as well as by the Animal Welfare Committee at the University of Rijeka, Faculty of Medicine and National Ethics Committee for the Protection of Animals Used for Scientific Purposes (#UP/I-322-01/18-01/30).

### Cytokine measurement in serum

Cytokine levels in serum samples were measured by polystyrene bead-based Luminex technology (R&D Systems) according to the manufacturer’s instructions. A Luminex 200 instrument (Luminex Corporation) was used to run the assay.

### Isolation and staining of brain leukocytes

Leukocytes from the brain were isolated using a standard protocol. Briefly, the brain was collected in RPMI 1640 with 3% (v/v) FCS and mechanically dissociated. A 30% Percoll/brain homogenate suspension was underlain with 70% Percoll in PBS and then centrifuged at 1050×*g* for 25 min. Cells in the interphase were collected for further analyses. Expression of MHC-II by microglia was determined by flow cytometry using the antibodies CD45.2-FITC (clone 104, dilution 1:100), CD11b-PE-Cy7 (clone M1/70, dilution 1:400), and MHC-II-APC (clone M5/114.15.2, dilution 1:200) purchased from Thermo Fisher. Microglia were gated as live CD45^int^CD11b^+^ cells.

### ELISA for MCMV-specific IgG

For the quantification of MCMV-specific IgG present in mouse serum samples collected at different times after MCMV infection, an indirect ELISA was performed. Briefly, MaxiSorp microtiter plates (Nunc) were coated with lysates of MCMV-infected cells or purified virions in ELISA binding buffer (0.1 M Na_2_HPO_4_, pH 9.0). Plates were incubated overnight at 4 °C. Non-specific binding sites were blocked with a solution of 10% (v/v) FCS in PBS for 1 h at room temperature. Wells were washed two times with ELISA wash buffer before samples were added and incubated for 90 min. The ELISA reaction was started after two washing steps by the use of peroxidase-labeled secondary antibodies (115-035-003, Dianova, dilution 1:5000) and TMB substrate. The reaction was stopped with 1 M H_2_SO_4_ before the absorbance was determined using a microplate multireader (Mithras LB 943; Berthold).

### IgG-dependent activation of the BW5147:FcγR-ζ reporter cells

The BW5147:FcγR-ζ reporter assays were performed as a surrogate test for FcγR activation^36,37^. Briefly, the assessment of IgG-dependent activation of the BW5147:FcγR-ζ cells was performed by incubating MCMV-infected cells with serial dilutions of serum in DMEM containing 10% (v/v) FCS for 30 min at 37 °C in an atmosphere of 5% CO_2_. To remove non-immune IgG, cells were washed three times with DMEM containing 10% (v/v) FCS before co-cultivation with BW5147:FcγR-ζ reporter cells was performed in RPMI containing 10% (v/v) FCS. The ratio between BW5147:FcγR-ζ and infected cells was 20:1. After 16 h of co-cultivation, supernatants were diluted 1:2 in ELISA sample buffer (PBS with 10% [v/v] FCS and 0.1% [v/v] Tween-20) and mIL-2 was measured by ELISA using the capture antibody JES6-1A12 and the biotinylated detection antibody JES6-5H4 (BD Pharmingen, dilution 1:500).

### Immunoblot analysis

Immunoblotting was performed by standard SDS PAGE followed by detection of indicated proteins using antibodies recognizing MCMV gB protein (15A12-H9) or GAPDH (sc-25778, Santa Cruz, dilution 1:5000). Proteins were visualized using peroxidase-coupled secondary antibodies (115-035-003 and 111-035-144, Dianova, dilution 1:10,000) and the ECL chemiluminescence system (Cell Signaling Technology). All blot samples shown together derive from the same experiment and were processed in parallel. Uncropped scans are provided in Supplementary Fig. 2.

### In vitro neutralization assay

To evaluate the neutralization effect of serum samples, an MCMV in vitro neutralization assay was conducted as described in ref. ^87^. Briefly, Δm157-MCMV:Luc was incubated with different dilutions of serum samples for 90 min prior to infection of fibroblasts. At 24 h post-infection, cells were lysed and luciferase activity was measured according to the manufacturer’s instructions (pjk) using a microplate luminometer (Mithras LB 943; Berthold). Neutralizing capacity was calculated with untreated Δm157-MCMV:Luc as reference.

### Statistical analysis

Statistical significance was determined using an unpaired *t*-test or Kruskal–Wallis test corrected for multiple comparisons as described in the figure legends. A *p* value of <0.05 was considered statistically significant. *p value <0.05. **p value <0.01. ***p value <0.001.

### Reporting summary

Further information on research design is available in the Nature Research Reporting Summary linked to this article.

## Supplementary information

Supplemental material

REPORTING SUMMARY

## Data Availability

All relevant data generated or analyzed during this study are included in this published article and its supplementary information files.

## Material availability

Further information and requests for resources and reagents should be directed to and will be fulfilled by the corresponding author, Mirko Trilling (Mirko.Trilling@uk-essen.de). All unique materials generated in this study are available from the corresponding author without restriction.
