# Supplementary material for: Maternal antibodies induced by a live attenuated vaccine protect neonatal mice from cytomegalovirus
Source: NPJ Vaccines. 2023 Feb 3;8:8. doi: 10.1038/s41541-023-00602-4 (PMC9898546; doi:10.1038/s41541-023-00602-4)
Supplement: Supplementary file 1 — Supplemental material [file 41541_2023_602_MOESM1_ESM.pdf]

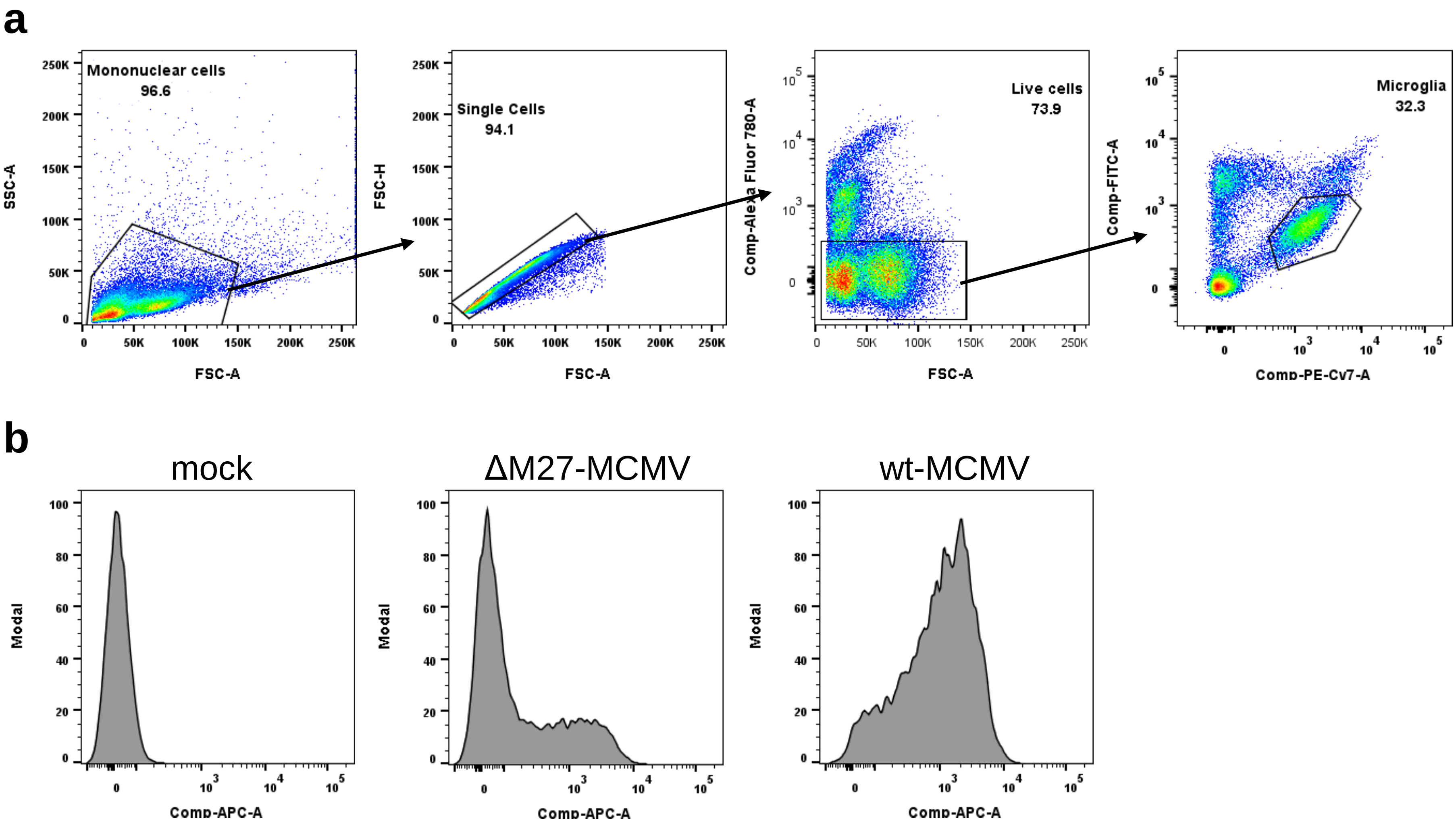

Supplementary Figure 1: Gating strategy for analysis of microglial MHC II expression
(a) Microglia were gated as indicated in the representative dot plots. To that aim, fixable viability dye (Alexa Fluor 780), anti-CD45-FITC and anti-CD11b-PE-Cy7 were used. (b) Representative microglial MHCII staining (anti-MHCII-APC) is shown for mock-infected, ΔM27-MCMV-infected and wt-MCMV-infected mice.

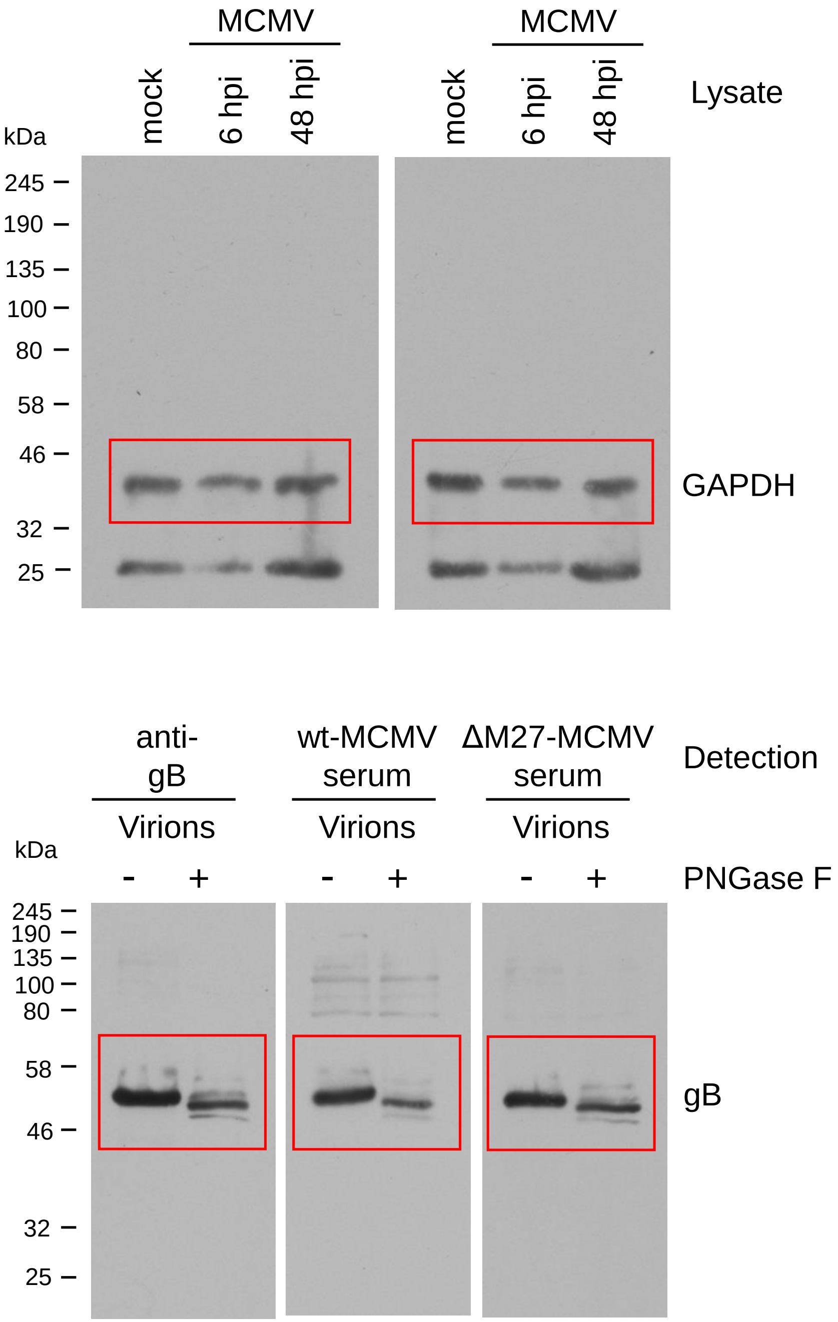

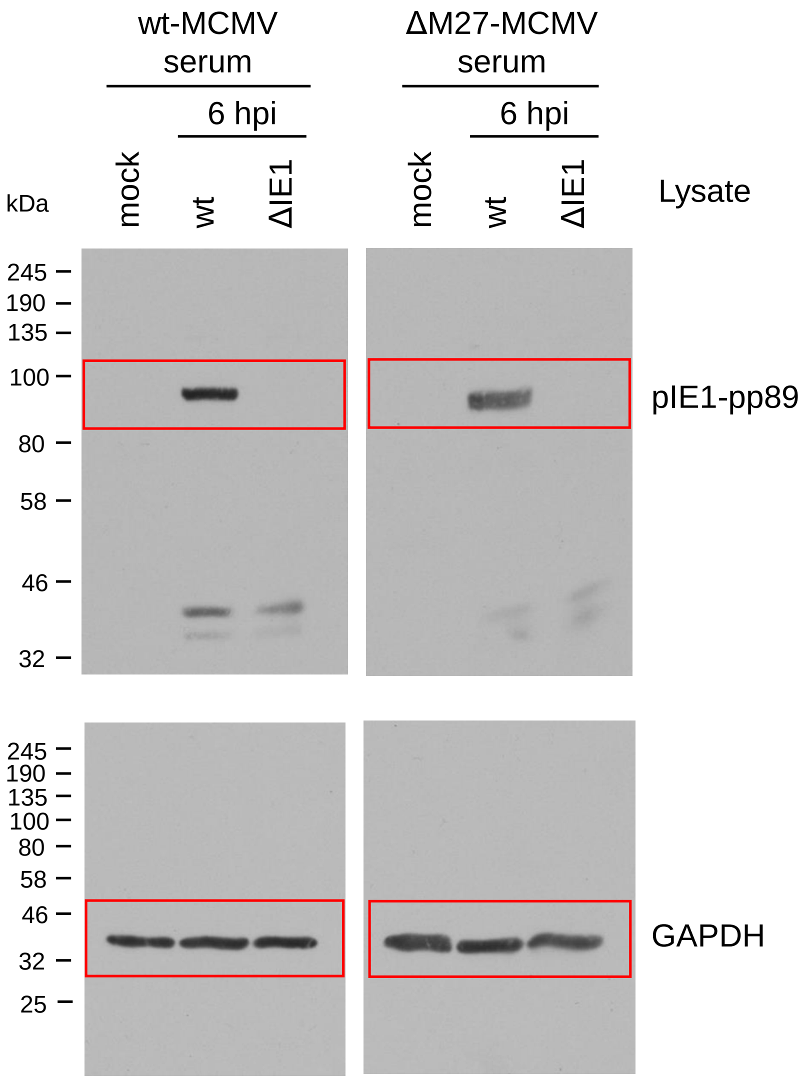

Supplementary Figure 2: Uncropped scans
Uncropped scans of immunoblots accompanied by the locations of molecular size markers.

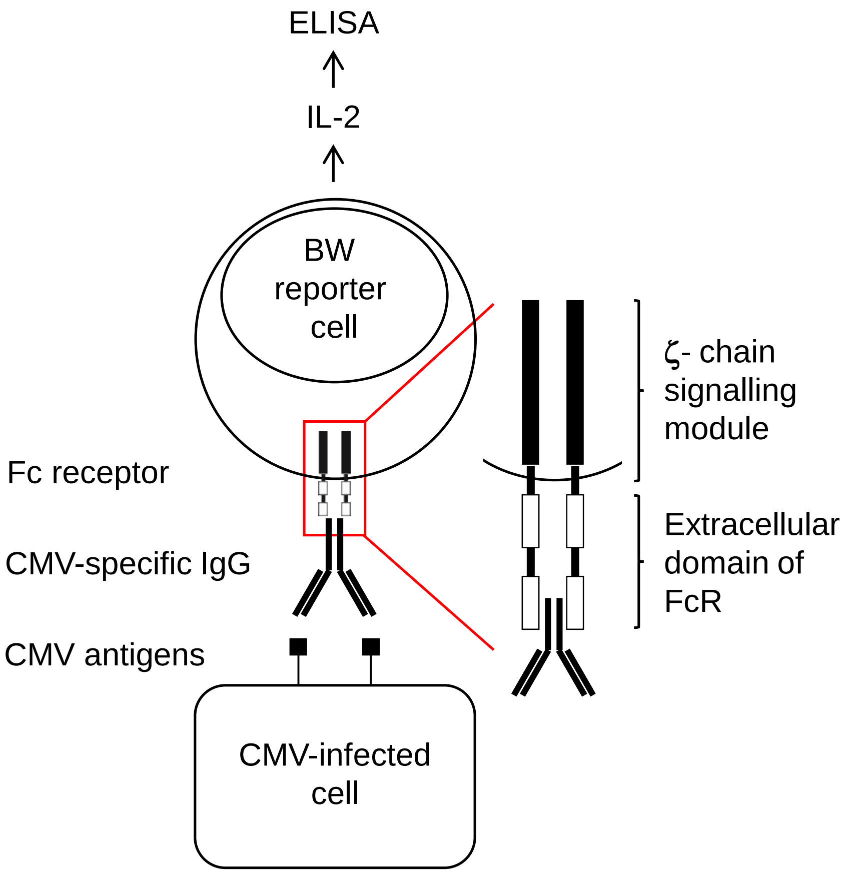

Supplementary Figure 3: A surrogate assay for Fcγ receptor activation
Schematic overview of the assay principle for the determination of FcγR responses. Reporter cells stably expressing chimeric FcγR molecules - composed of the extracellular IgG-binding domain of the FcγR fused to the CD3ζ-chain as intracellular signaling module - were used. The activation of the chimeric FcγRs by antigen-bound IgG leads to IL-2 secretion, which can be quantified by ELISA.

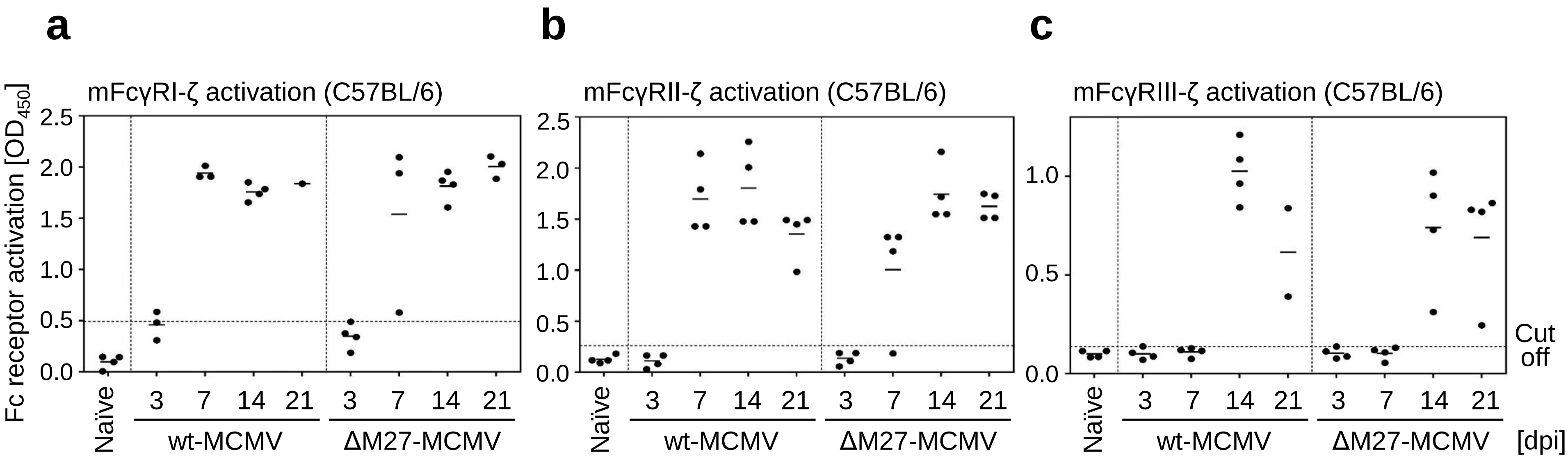

Supplementary Figure 4: Immunization of C57BL/6 mice with ΔM27-MCMV raises strong MCMV-specific FcγR-activating IgG responses

C57BL/6 mice were infected i.p. with wt-MCMV or ΔM27-MCMV. At 3, 7, 14, and 21 dpi, serum samples were collected. FcγR-activating capacity was quantified from sera by mIL-2 ELISA using a surrogate assay as described in the Methods section. The mean values as well as the values of individual mice (n=4 per group) are shown. (a) Activation assay for mFcγRI. (b) Activation assay for mFcγRII. (c) Activation assay for mFcγRIII.

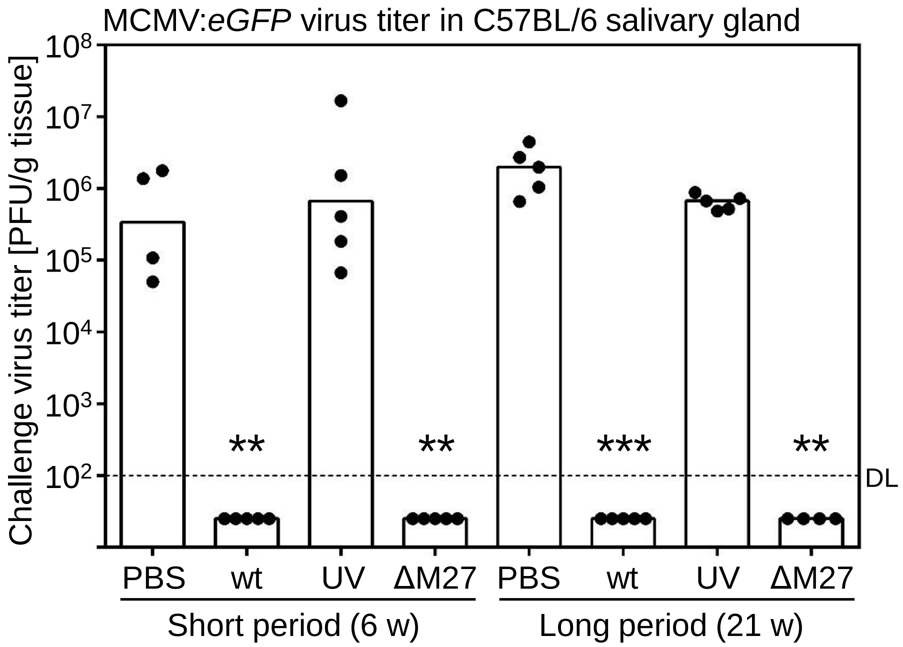

Supplementary Figure 5: Vaccination with ΔM27-MCMV protects adult mice from challenge infections

C57BL/6 mice were vaccinated with $2*10^5$ PFU of wt-MCMV, ΔM27-MCMV, or UV-inactivated wt-MCMV. At 6 and 21 weeks post vaccination, mice were challenged with $2*10^5$ PFU of MCMV:eGFP. At 21 days post challenge infection, salivary glands were harvested and frozen. The MCMV:eGFP titers were determined from organ homogenates by plaque titration. All titrations were done in quadruplicate. Bars depict the geometric mean, dots show titers of individual mice (n=4-5 per group). DL, detection limit. All vaccinated groups were compared to the corresponding control group by Kruskal-Wallis test corrected for multiple comparisons by controlling the false discovery rate. **, p value <0.01. ***, p value <0.001.

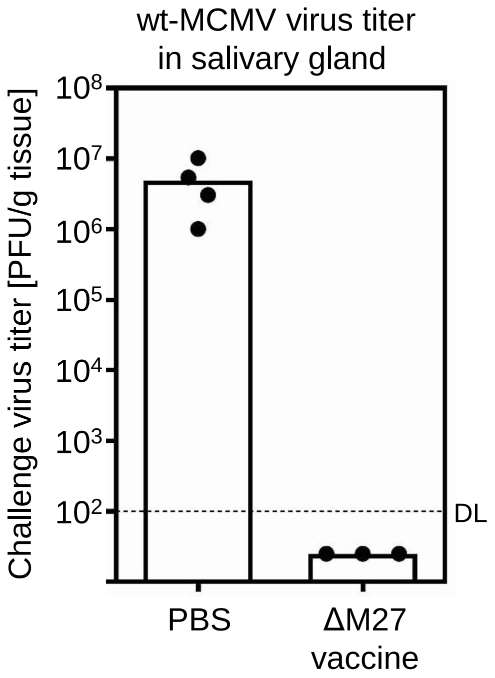

Supplementary Figure 6: Vaccination with ΔM27-MCMV protects adult mice from homotypic challenge infections

The experiment was conducted as described in Fig. 7, but wt-MCMV instead of MCMV:eGFP was used for the challenge infection.

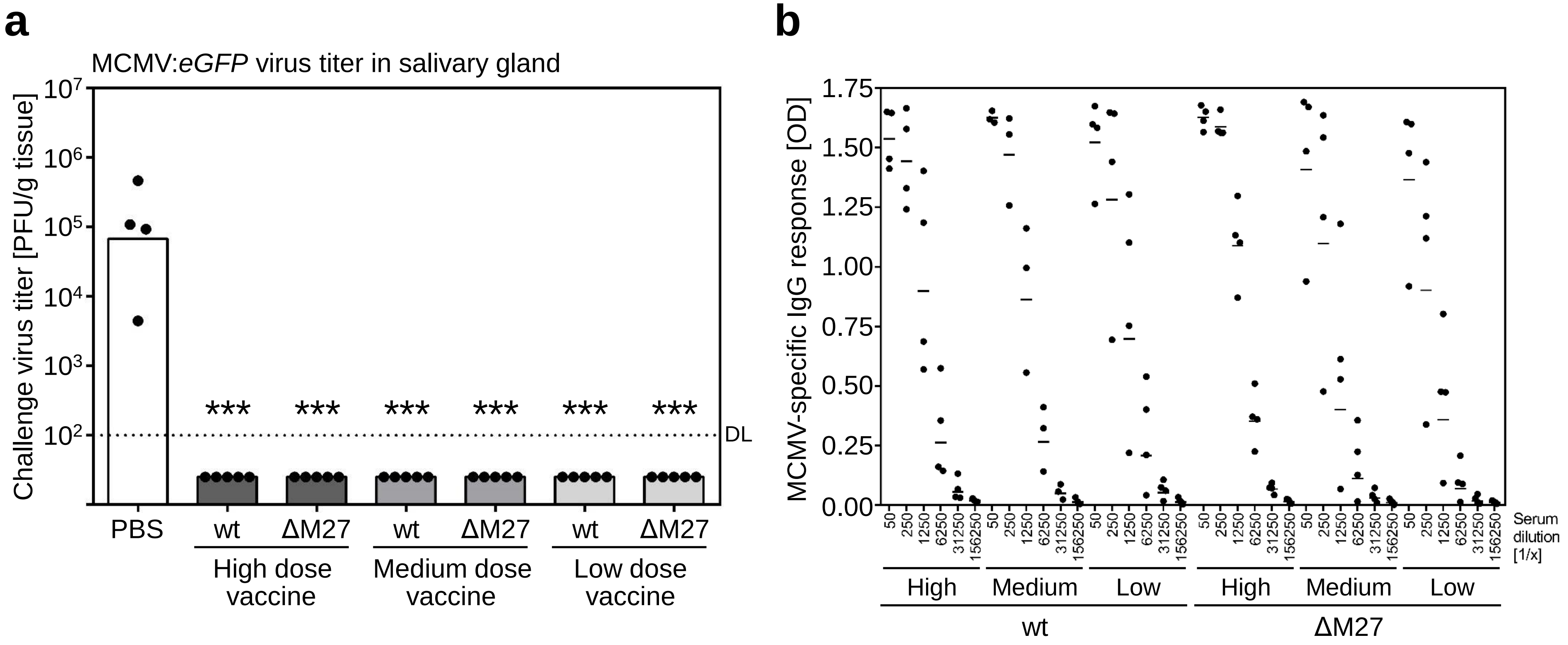

Supplementary Figure 7: Vaccination with low doses of ΔM27-MCMV protects adult mice from challenge infections

(a) BALB/c mice were vaccinated with $2*10^5$ PFU (high dose), $4*10^4$ PFU (medium dose), and $8*10^3$ PFU (low dose) of wt-MCMV or ΔM27-MCMV. At 6 weeks post vaccination, mice were challenged with $2*10^5$ PFU of MCMV:eGFP. At 21 days post challenge infection, salivary glands were harvested and frozen. The MCMV:eGFP titers were determined from organ homogenates by plaque titration. Bars depict the geometric mean, dots show titers of individual mice. DL, detection limit. All vaccinated groups were compared to the corresponding control group by Kruskal-Wallis test corrected for multiple comparisons by controlling the false discovery rate. **, p value <0.01. ***, p value <0.001. (b) BALB/c mice were infected i.p. with wt-MCMV or ΔM27-MCMV as in (a). At 6 weeks post-infection, serum samples were collected. MCMV-specific IgG antibodies recognizing MCMV-infected cell proteins were quantified by ELISA using indicated serum dilutions. The geometric mean values (horizontal bars) as well as the values of individual mice are shown.
