# Supplementary material for: Maternal antibodies induced by a live attenuated vaccine protect neonatal mice from cytomegalovirus
Source: NPJ Vaccines. 2023 Feb 3;8:8. doi: 10.1038/s41541-023-00602-4 (PMC9898546; doi:10.1038/s41541-023-00602-4)
Supplement: Supplementary file 2 — REPORTING SUMMARY [file 41541_2023_602_MOESM2_ESM.pdf]

nature portfolio

Corresponding author(s): Mirko Trilling

Last updated by author(s): Dec 5, 2022

Reporting Summary

Nature Portfolio wishes to improve the reproducibility of the work that we publish. This form provides structure for consistency and transparency in reporting. For further information on Nature Portfolio policies, see our Editorial Policies and the Editorial Policy Checklist.

Statistics

For all statistical analyses, confirm that the following items are present in the figure legend, table legend, main text, or Methods section.

n/a	Confirmed	
☐	☒	The exact sample size (*n*) for each experimental group/condition, given as a discrete number and unit of measurement
☐	☒	A statement on whether measurements were taken from distinct samples or whether the same sample was measured repeatedly
☐	☒	The statistical test(s) used AND whether they are one- or two-sided<br>*Only common tests should be described solely by name; describe more complex techniques in the Methods section.*
☒	☐	A description of all covariates tested
☐	☒	A description of any assumptions or corrections, such as tests of normality and adjustment for multiple comparisons
☐	☒	A full description of the statistical parameters including central tendency (e.g. means) or other basic estimates (e.g. regression coefficient) AND variation (e.g. standard deviation) or associated estimates of uncertainty (e.g. confidence intervals)
☐	☒	For null hypothesis testing, the test statistic (e.g. *F*, *t*, *r*) with confidence intervals, effect sizes, degrees of freedom and *P* value noted<br>*Give P values as exact values whenever suitable.*
☒	☐	For Bayesian analysis, information on the choice of priors and Markov chain Monte Carlo settings
☒	☐	For hierarchical and complex designs, identification of the appropriate level for tests and full reporting of outcomes
☒	☐	Estimates of effect sizes (e.g. Cohen's *d*, Pearson's *r*), indicating how they were calculated

Our web collection on statistics for biologists contains articles on many of the points above.

Software and code

Policy information about availability of computer code

Data collection	Data collection was performed by use of the microplate multireader Mithras2 LB 943 and MicroWin software (Berthold Technologies) as well as a Luminex 200 instrument and the corresponding software (Luminex Corporation).
Data analysis	The results of the experiments were analyzed using Excel 2016 and GraphPad Prism 8 software.

For manuscripts utilizing custom algorithms or software that are central to the research but not yet described in published literature, software must be made available to editors and reviewers. We strongly encourage code deposition in a community repository (e.g. GitHub). See the Nature Portfolio guidelines for submitting code & software for further information.

Data

Policy information about availability of data

All manuscripts must include a data availability statement. This statement should provide the following information, where applicable:

- Accession codes, unique identifiers, or web links for publicly available datasets
- A description of any restrictions on data availability
- For clinical datasets or third party data, please ensure that the statement adheres to our policy

All relevant data generated or analysed during this study are included in this published article and its supplementary information files.

Human research participants

Policy information about studies involving human research participants and Sex and Gender in Research.

Reporting on sex and gender	*Use the terms sex (biological attribute) and gender (shaped by social and cultural circumstances) carefully in order to avoid confusing both terms. Indicate if findings apply to only one sex or gender; describe whether sex and gender were considered in study design whether sex and/or gender was determined based on self-reporting or assigned and methods used. Provide in the source data disaggregated sex and gender data where this information has been collected, and consent has been obtained for sharing of individual-level data; provide overall numbers in this Reporting Summary. Please state if this information has not been collected. Report sex- and gender-based analyses where performed, justify reasons for lack of sex- and gender-based analysis.*
Population characteristics	*Describe the covariate-relevant population characteristics of the human research participants (e.g. age, genotypic information, past and current diagnosis and treatment categories). If you filled out the behavioural & social sciences study design questions and have nothing to add here, write "See above."*
Recruitment	*Describe how participants were recruited. Outline any potential self-selection bias or other biases that may be present and how these are likely to impact results.*
Ethics oversight	*Identify the organization(s) that approved the study protocol.*

Note that full information on the approval of the study protocol must also be provided in the manuscript.

Field-specific reporting

Please select the one below that is the best fit for your research. If you are not sure, read the appropriate sections before making your selection.

☒ Life sciences ☐ Behavioural & social sciences ☐ Ecological, evolutionary & environmental sciences

For a reference copy of the document with all sections, see nature.com/documents/nr-reporting-summary-flat.pdf

Life sciences study design

All studies must disclose on these points even when the disclosure is negative.

Sample size	Samples sizes of 4-6 mice are common and well accepted practice in MCMV infection experiments.
Data exclusions	No data were excluded.
Replication	The findings have been independently reproduced (e.g., regarding neutralizing antibody responses [Fig. 5 and 7C] and FcγR activation [Fig. 6 and Suppl.-Fig. 4]) in parts by different researchers and in different laboratories. Importantly, immune responses and protection were evident in different mouse strains (BALB/c and C57BL/6), against different challenge viruses (Fig. 7 & Suppl.-Fig. 6), after different periods following vaccination (Fig. 7B and Suppl.-Fig. 5, left versus right panel), and after different vaccine doses (Suppl.-Fig. 7). Protective effects were verified in various organs such as brain, lung, liver, spleen, and/or salivary gland (see Fig. 8 and 9). ELISA-reactive antibody responses were consistent using infected cells or purified virions as targets (Fig. 2 & 3). FcγR activation was consistent across all 4 Fcγ receptors.
Randomization	Allocation of mice to groups was random (keeping characteristics such as sex, age, and weight equal across groups).
Blinding	Blinding was impossible because the responsible investigator had to ensure the welfare of mice, and confirm compliance with all regulatory requirements for all animal experiment procedures. However, at various instances, technical staff members, who were not directly involved in the project, determined the virus titers or the immune responses as an unbiased service.

Reporting for specific materials, systems and methods

We require information from authors about some types of materials, experimental systems and methods used in many studies. Here, indicate whether each material, system or method listed is relevant to your study. If you are not sure if a list item applies to your research, read the appropriate section before selecting a response.

Materials & experimental systems

n/a	Involved in the study
☐	☒ Antibodies
☐	☒ Eukaryotic cell lines
☒	☐ Palaeontology and archaeology
☐	☒ Animals and other organisms
☒	☐ Clinical data
☒	☐ Dual use research of concern

Methods

n/a	Involved in the study
☒	☐ ChIP-seq
☐	☒ Flow cytometry
☒	☐ MRI-based neuroimaging

Antibodies

Antibodies used	CD45.2-FITC (clone 104) ,Thermo Fisher # MCD45201<br>CD11b-PE-Cy7 (clone M1/70), Thermo Fisher # 25-0112-82<br>MHC-II-APC (clone M5/114.15.2), Thermo Fisher # 17-5321-82<br>mouse IL-2 ELISA capture and detection antibody, JES6-1A12 and JES6-5H4, BD Pharmingen # 554424 and # 554426<br>MCMV gB (15A12-H9)<br>GAPDH (sc-25778, Santa Cruz)
Validation	The commercially available antibodies were validated by the manufacturer.

Eukaryotic cell lines

Policy information about cell lines and Sex and Gender in Research

Cell line source(s)	BW5147:FcγR-ζ reporter cells were obtained from Hartmut Hengel.
Authentication	The authenticity of the reporter cells was confirmed by sequencing of the reporter cassette.
Mycoplasma contamination	All cells were regularly tested for mycoplasm contamination and discarded if positive.
Commonly misidentified lines (See ICLAC register)	Not applicable

Animals and other research organisms

Policy information about studies involving animals; ARRIVE guidelines recommended for reporting animal research, and Sex and Gender in Research

Laboratory animals	Mus musculus, strain C57BL/6 and BALB/c, both male and female mice were included, experiments involved newborn and adult mice.
Wild animals	The study did not involve wild animals.
Reporting on sex	The study involved male and female mice. No sex-related differences were observed for the vaccine efficiency in adult animals. Obviously, maternal antibody responses were only transmitted by female mice.
Field-collected samples	The study did not involve samples collected from the field.
Ethics oversight	All procedures were performed in accordance with the guidelines of the University Hospital Essen, Germany, the national animal protection law (Tierschutzgesetz [TierSchG]) and the recommendations of the Federation of European Laboratory Animal Science Association (FELASA). The study was approved by the Northrhine-Westphalia State Office for Nature, Environment and Consumer Protection (LANUV NRW, Düsseldorf, Northrhine-Westphalia, Germany; permit #84-02.04.2014.A390 for MEF preparation and #84-02.04.2013.A414 for MCMV in vivo infections of adult mice) as well as by the Animal Welfare Committee at the University of Rijeka, Faculty of Medicine and National ethics Committee for the Protection of Animals Used for Scientific Purposes (#UP/I-322-01/18-01/30).

Note that full information on the approval of the study protocol must also be provided in the manuscript.

Flow Cytometry

Plots

Confirm that:

☐ The axis labels state the marker and fluorochrome used (e.g. CD4-FITC).

☐ The axis scales are clearly visible. Include numbers along axes only for bottom left plot of group (a 'group' is an analysis of identical markers).

☐ All plots are contour plots with outliers or pseudocolor plots.

☐ A numerical value for number of cells or percentage (with statistics) is provided.

Methodology

Sample preparation	Mice were perfused with cold PBS and each brain was collected in RPMI 1640 with 3% FCS and mechanically dissociated. A 30% Percoll/brain homogenate suspension was underlaid with 70% Percoll in PBS and then centrifuged at 1050 g for 25 min. Cells in the interphase were collected for further analysis. Following isolation, mononuclear cells were labeled with antibodies.
Instrument	BD FACSAria IIu
Software	FlowJo v10 (Tree Star, Inc.)
Cell population abundance	No cell sorting was performed.
Gating strategy	Mononuclear cells were identified based on forward and side scatter properties and live cells were gated by using fixable viability dye. Microglia were identified as live CD11b+CD45low cells. To that aim, anti-mouse CD45, CD11b, MHC-II, and Fixable Viability Dye (Thermo Fischer Scientific) were used.

☒ Tick this box to confirm that a figure exemplifying the gating strategy is provided in the Supplementary Information.
